# Synthesis of ordered carbonaceous frameworks from organic crystals

**DOI:** 10.1038/s41467-017-00152-z

**Published:** 2017-07-24

**Authors:** Hirotomo Nishihara, Tetsuya Hirota, Kenta Matsuura, Mao Ohwada, Norihisa Hoshino, Tomoyuki Akutagawa, Takeshi Higuchi, Hiroshi Jinnai, Yoshitaka Koseki, Hitoshi Kasai, Yoshiaki Matsuo, Jun Maruyama, Yuichiro Hayasaka, Hisashi Konaka, Yasuhiro Yamada, Shingi Yamaguchi, Kazuhide Kamiya, Takuya Kamimura, Hirofumi Nobukuni, Fumito Tani

**Affiliations:** 1 Institute of Multidisciplinary Research for Advanced Materials, Tohoku University, 2−1−1 Katahira, Aoba, Sendai 980−8577 Japan; 2 PRESTO, the Japan Science and Technology Agency (JST), 4-1-8 Honcho, Kawaguchi, 332-0012 Japan; 3 Department of Materials Science and Chemistry, Graduate School of Engineering, University of Hyogo, 2167 Shosha Himeji, Hyogo, 671-2280 Japan; 4 Research Division of Environmental Technology, Osaka Research Institute of Industrial Science and Technology, 1-6-50, Morinomiya, Joto-ku, Osaka 536-8553 Japan; 5 The Electron Microscopy Centre, Tohoku University, 2−1−1 Katahira, Aoba, Sendai 980−8577 Japan; 6 Application & Software Development Department, X-ray Instrument Divistion, Rigaku Corporation, 3-9-12 Matsubara-cho, Akishima-shi, Tokyo 196-8666 Japan; 7 Graduate School of Engineering, Chiba University, 1-33 Yayoi, Inage, Chiba 263-8522 Japan; 8 Department of Applied Chemistry, The University of Tokyo, 7-3-1 Hongo, Bunkyo-ku, Tokyo 113-8656 Japan; 9 Research Center for Solar Energy Chemistry, Osaka University, 1-3 Machikaneyama, Toyonaka, Osaka 560-8531 Japan; 10 Institute for Materials Chemistry and Engineering, Kyushu University, 744 Motooka, Nishi-ku, Fukuoka 819-0395 Japan

## Abstract

Despite recent advances in the carbonization of organic crystalline solids like metal-organic frameworks or supramolecular frameworks, it has been challenging to convert crystalline organic solids into ordered carbonaceous frameworks. Herein, we report a route to attaining such ordered frameworks via the carbonization of an organic crystal of a Ni-containing cyclic porphyrin dimer (Ni_2_-CPD_Py_). This dimer comprises two Ni–porphyrins linked by two butadiyne (diacetylene) moieties through phenyl groups. The Ni_2_-CPD_Py_ crystal is thermally converted into a crystalline covalent-organic framework at 581 K and is further converted into ordered carbonaceous frameworks equipped with electrical conductivity by subsequent carbonization at 873–1073 K. In addition, the porphyrin’s Ni–N_4_ unit is also well retained and embedded in the final framework. The resulting ordered carbonaceous frameworks exhibit an intermediate structure, between organic-based frameworks and carbon materials, with advantageous electrocatalysis. This principle enables the chemical molecular-level structural design of three-dimensional carbonaceous frameworks.

## Abstract (web summary)

Carbon-based materials are promising alternatives to noble metal catalysts, but their structures are typically disordered and difficult to control. Here, the authors obtain ordered carbonaceous frameworks with advantageous electrocatalytic properties via the carbonization of nickel-containing porphyrin dimer networks.

## Introduction

Carbonaceous materials are generally prepared by carbonization of organic substances. During the carbonization process, organic precursors are thermally converted into aggregations of imperfect graphene fragments via intermediates of polycyclic aromatic compounds^1^. Despite their complicated and random structures, carbonaceous materials possess many advantageous properties (electrical conductivity, chemical and thermal stability, light weight). Hence, they are used in a variety of applications including adsorbents, catalysts, supercapacitors, and polymer-electrolyte fuel cells (PEFCs)^2^. When precursors like porphyrins and phthalocyanines with metal/nitrogen (M/N) are employed, their heteroatoms are dispersed in the resulting M/N/C composites^3^. Thus, they show great potential as non-Pt catalysts for oxygen-reduction reactions in PEFCs^4–6^. The thermal conversion process for the production of these carbonaceous materials comprises complex, poorly controlled radical reactions^1^. Hence, the molecular-level control of this process to realize next-generation, high-performance functional carbonaceous materials is challenging. To overcome this, carbonization of molecular-based crystals (metal-organic frameworks^7–16^/molecular organic crystals^17, 18^) with well-designed chemical and supramolecular structures has been employed. This controls the process indirectly via the chemical structures of the precursors while retaining the bulk particle morphology^8, 10^ and/or approximate porosity^17^, even after carbonization. However, molecular-based crystals convert into intrinsically amorphous carbonaceous frameworks and the precursor structure and molecular features are totally lost during carbonization. Structure-preserving carbonization has only been achieved in mesoscopic organic structures (>5 nm) formed by self-assembly of block-copolymers or surfactant templates^19–21^. Moreover, the direct conversion of organic crystals into ordered carbonaceous frameworks (OCFs) has not been demonstrated.

Herein, we propose the design and supramolecular network structure of a precursor molecule. Our aim was to preserve the precursor structure, while converting a limited part into a carbonaceous framework, to synthesize hybrid materials. These are equipped with precursor structural and chemical features and carbon material properties. The cyclic porphyrin dimer (Ni_2_-CPD_Py_)^22^ met these criteria. Ni_2_-CPD_Py_ comprises two Ni-porphyrins linked by two butadiyne (diacetylene) moieties through phenyl groups; each porphyrin includes two *meso*-pyridyl groups. The M–N_4_ (M = metal) unit in the porphyrin ring is thermally stable (~973 K)^23, 24^. Diacetylene is thermally polymerized to poly(diacetylene) to form a rigid crosslinked network, allowing the precursor morphology to be maintained during carbonization. Ni_2_-CPD_Py_ does not contain volatile fragments^1^, thus, a high carbon yield essential to retain the overall framework morphology of the precursor crystal is predicted.

## Results

### Carbonization of Ni_2_-CPD_Py_

**Fig. 1:**
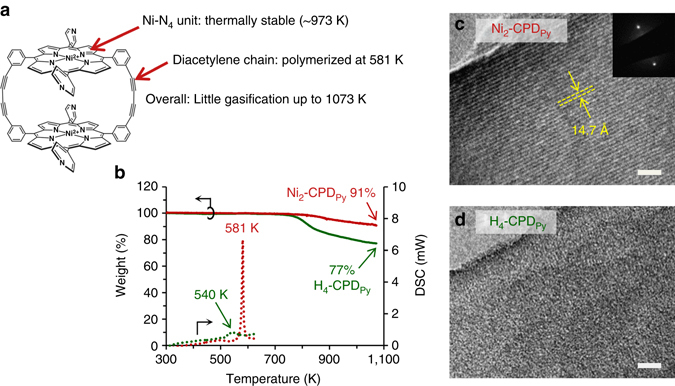
**Structure of Ni**_**2**_ **-CPD**_**Py**_ **and its thermal properties up to a temperature of 1073 K. a** Structure of Ni_2_-CPD_Py_ and its superior properties as a precursor of carbonization. **b** TG (*solid lines*) and DSC curves (*dotted lines*) of Ni_2_-CPD_Py_ (*red*) and H_4_-CPD_Py_ (*green*). Yields at 1073 K are described for TG curves, while peak temperatures are shown for DSC curves. **c**, **d** TEM images of the residues of (**c**) Ni_2_-CPD_Py_ and (**d**) H_4_-CPD_Py_ after TG measurements. Scale bars = 10 nm. Inset: a selected-area diffraction pattern for **c**

The molecular structure of Ni_2_-CPD_Py_ is shown in Fig. 1a. The thermal behaviour of Ni_2_-CPD_Py_ was investigated and compared to the corresponding free base porphyrin (H_4_-CPD_Py_)^25^. Figure 1b displays weight changes of Ni_2_-CPD_Py_ and H_4_-CPD_Py_ measured by thermogravimetry (TG) in N_2_ and their differential scanning calorimetry (DSC) curves. The molecules do not exhibit weight loss for temperatures ≤750 K, demonstrating excellent heat-stability and minimal volatility. Thus, Ni_2_-CPD_Py_ and H_4_-CPD_Py_ afforded high yields (91% and 77%, respectively, 1073 K). Their carbonization processes were further analysed by temperature-programmed desorption (TPD) with thermogravimetry/photoionization mass spectrometry (TG-PI-MS, Supplementary Fig. 1). Few species (C_6_H_6_, C_5_NH_5_, C_7_H_8_, C_4_NH_5_/C_5_H_7_, C_5_H_8_, and NH_3_) were desorbed from Ni_2_-CPD_Py_, while a variety of species were found in H_4_-CPD_Py_, suggesting that limited decomposition occurs in Ni_2_-CPD_Py_. The carbonaceous residues afforded after TG measurement were analysed by transmission electron microscopy (TEM, Fig. 1c,d). The product derived from Ni_2_-CPD_Py_ displayed an ordered structure (periodicity = 14.7 Å). The corresponding electron diffraction pattern clearly differed from that of the graphite (002) plane (periodicity = 3.4 Å). Conversely, the H_4_-CPD_Py_ residue did not exhibit a highly ordered structure (Fig. 1d). Thus, the porphyrin cation significantly affected the carbonization process and the resulting structure. Ni stabilizes the porphyrin against the thermochemical decomposition, thereby achieving the better yield and retaining the ordered structure.

The Ni_2_-CPD_Py_ DSC curve (Fig. 1b) exhibits an intense exothermic peak at 581 K (integration = 142 J g^−1^ heat). This corresponds to 102 kJ mol^−1^ per diacetylene amount included in Ni_2_-CPD_Py_ and this value is ascribed mainly to the diacetylene heat of polymerization (well in agreement with literature values: 80–151 kJ mol^−1^)^26, 27^. The H_4_-CPD_Py_ DSC curve exhibits a weaker peak at 540 K, affording 41 kJ mol^−1^ per diacetylene. This suggests that the cross-linking in H_4_-CPD_Py_ is not well developed, resulting in the collapse of the ordered structure (Fig. 1d).

### Crystallographic structural changes upon carbonization

**Fig. 2:**
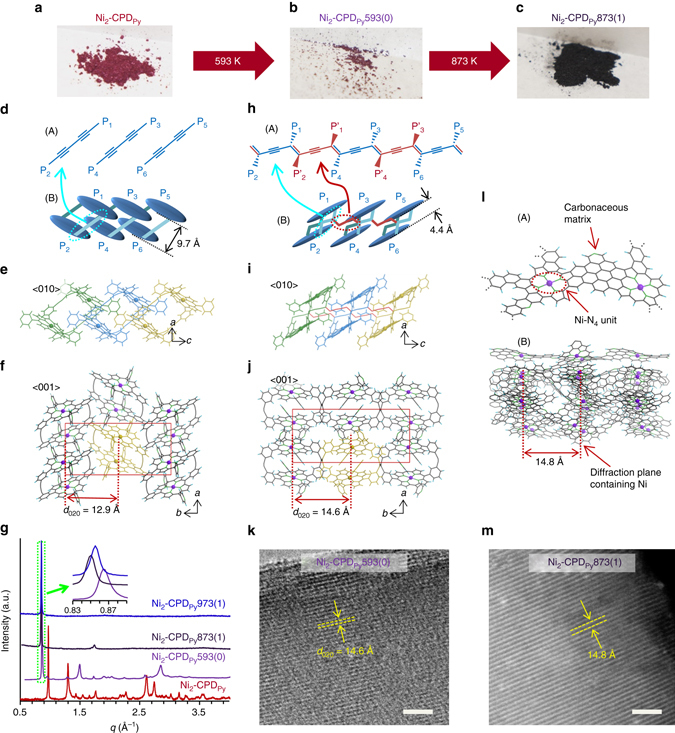
Structure evolution of Ni_2_-CPD_Py_ upon heat-treatment. **a**–**c** Photographs of (**a**) Ni_2_-CPD_Py_, (**b**) Ni_2_-CPD_Py_593(0), and (**c**) Ni_2_-CPD_Py_873(1). **d** Schematic representation of diacetylene chains (A) and Ni_2_-CPD_Py_ molecules (B); P_*n*_ = porphyrin unit (*n* = 1–6). **e** Molecular structure of **d**-(B): bottom illustration, <010> direction; each molecule is displayed in a different colour. **f** Larger area view: <001> direction; yellow part corresponds to the yellow moiety in **e**. **g** PXRD patterns of Ni_2_-CPD_Py_ and heat-treated samples; inset: enlarged intense peaks of heat-treated samples. **h**–**j** Ni_2_-CPD_Py_593(0) packing structure. **h** Schematic representation of part corresponding to **d**. In (A), porphyrins locate next to those shown in (B) are indicated with the symbol, P_*n*_’. **i** Molecular structure of **h**-(B): <010> direction. Coloured moieties correspond to those in **e**. **j** Larger area view from the <001> direction; yellow part corresponds to the yellow moiety in **i**. Red box in **f** and **j** is unit cell. **k** TEM image of Ni_2_-CPD_Py_593(0). **l** Expected molecular-level structure of Ni_2_-CPD_Py_873(1); an enlarged part (A) and a larger region (B) corresponding to **j**. For **f**, **j**, and **l**: C, H, N, O, Ni = *black*, *light blue*, *green*, *red*, and *purple*, respectively. **m** HAADF-STEM image of Ni_2_-CPD_Py_873(1). Scale bars in **k** and **m** are 10 nm

The structure evolution of Ni_2_-CPD_Py_ upon heat treatment was analysed to understand the formation mechanism of the fine-ordered structure (Fig. 1c). Figure 2 summarizes the characterization results of Ni_2_-CPD_Py_ and its heat-treated samples. Ni_2_-CPD_Py_ almost retains its colour after polymerization [Ni_2_-CPD_Py_593(0)], indicating the preservation of the porphyrin unit (Fig. 2a,b). After carbonization, the sample turned black (Fig. 2c), confirming the conversion into a carbonaceous substance (graphene sheet formation). We previously reported the single-crystal structure of Ni_2_-CPD_Py_ accommodating toluene as a guest^22^. Herein, we employed a guest-free crystal (Fig. 2d–f) as a precursor to exclude the effect of toluene and simplify the carbonization process. The crystallographic structure was solved by the direct space method^28–30^ and Rietveld refinement^31^ of the powder X-ray diffraction (PXRD) pattern (Fig. 2g and Supplementary Fig. 2). The Ni_2_-CPD_Py_ molecule exhibits a slipped conformation (Fig. 2d–f). This differed from the overlapped conformation of its single crystal form^22^, and the distance between the two porphyrins was determined as 9.7 Å. The Ni_2_-CPD_Py_ molecules are aligned along the *c*-axis to form columnar arrangement (Fig. 2e), and the columns are integrated to form the structure shown in Fig. 2f (see the detail structure in Supplementary Fig. 3 and Supplementary Movie 1). The diacetylene moieties are located in both sides of the column along the *b* axis. This ordered arrangement enables solid-phase polymerization to form another crystalline phase (Fig. 2h–j) that was similarly solved (Supplementary Fig. 4). Due to polymerization, the distance between the two porphyrins shortens (4.4 Å) and the Ni_2_-CPD_Py_ molecules along the *b* and *c* axes are cross-linked through a poly(diacetylene) backbone to form a two-dimensional sheet. These sheets are stacked to form a crystal structure (Fig. 2j and Supplementary Fig. 5, Supplementary Movie 2). The polymerization of diacetylene moieties into poly(diacetylene) backbone (Fig. 2d, h) is commonly observed in organic molecules^26, 32^. Solid ^13^C NMR confirmed that the diacetylene moieties are almost completely cross-linked to form the poly(diacetylene) form (Supplementary Fig. 6). Thus, Ni_2_-CPD_Py_593(0) is insoluble in chloroform (good solvent for Ni_2_-CPD_Py_) and ^1^H NMR and matrix-assisted laser desorption ionization time-of-flight mass spectrometry (MALDI-TOF-MS) did not detect any remaining monomer/oligomers. The transformation from monomer crystal into a crystalline covalent-organic framework is ascribed to the close proximity of the diacetylene moieties in Ni_2_-CPD_Py_ (Fig. 2d–f), allowing solid-phase polymerization to proceed. On the other hand, H_4_-CPD_Py_ is not a highly crystalline solid, and its packing structure cannot be solved from the PXRD pattern in its guest-free form (Supplementary Fig. 7). H_4_-CPD_Py_ has broad PXRD peaks, and it means that the solid contains irregular packing structures and distributed distances between diacetylene moieties, causing imperfect polymerization. Thus, the original packing structure collapses during pyrolysis (Supplementary Fig. 7).

Upon polymerization, the *d*-spacing (12.9 Å) of the original Ni_2_-CPD_Py_ (020) plane increases to 14.6 Å (Fig. 2g) and its periodicity is clearly observed in the TEM image (Fig. 2k). The structure regularity of the (020) plane is well retained, even after heat treatment (873 K). This is confirmed by the sharp peak (inset of Fig. 2g; *d*-spacing = 14.8 Å) that exhibits a *d*-spacing that is slightly greater than that of its precursor polymer (14.6 Å). Generally, the carbonization of organic substances results in matrix shrinkage^19, 20^ because graphene and its stacked graphitic structure have atomically denser frameworks. Thus, the increase in *d*-spacing indicates the formation of low-density framework structures. The resulting Ni_2_-CPD_Py_873(1) was further analysed by high angle annular dark-field scanning transmission electron microscopy (HAADF-STEM; Fig. 2m: white-coloured area = Ni atoms). Ni rarely forms nanoparticles or aggregates often generated in the carbonaceous residues of metal porphyrins^33, 34^ (an example is shown later in the carbonization of 5,10,15,20-tetraphenyl-21H,23H-porphine nickel(II) [Ni-TPP]). Ni and NiO formation was not detected, even by synchrotron PXRD analysis (Supplementary Fig. 8). The image in Fig. 2m well agrees with the TEM image (Supplementary Fig. 9). Notably, the ordered structure spreads to several hundred nanometers (Supplementary Fig. 9). The results of TEM, HAADF-STEM, and synchrotron PXRD reveal that Ni is not aggregated as Ni metal or NiO, and exists along the structure regularity derived from the (020) plane of its precursor. As shown later, Ni retains its original coordination structure (Ni-N_4_) in the carbonaceous framework. Moreover, synchrotron PXRD (Supplementary Fig. 8) and fast Fourier transform TEM images (Supplementary Fig. 10) prove the presence of several diffraction planes other than the (020) plane and high-order planes in Ni_2_-CPD_Py_873(1). This indicates its well-ordered structure, similar to organic-based frameworks. Fig. 2l displays a possible Ni_2_-CPD_Py_873(1) framework built on experimental evidence (see Supplementary Methods about the details of model construction). Porphyrin moieties are linked by a carbonaceous matrix keeping the Ni–N_4_ structure and their approximate original positions [Ni_2_-CPD_Py_593(0)]. Thus, Ni atoms form the diffraction plane observed by PXRD and TEM/STEM analysis. As represented by this model (3D view is provided in Supplementary Movie 3), Ni_2_–CPD_Py_873(1) exhibits an intermediate framework between an organic substance and carbon, making this material distinguishable from any other substances. Moreover, the sample carbonized at 973 K still retains structural regularity with very small shrinkage (Fig. 2g, *d*-spacing = 14.7 Å) confirming the excellent heat stability of the ordered structure.

### Chemical structure transition upon carbonization

The change in elemental composition associated with carbonization is summarized in Table 1. The amount of hydrogen decreases with an increase in temperature suggesting growth of the graphene sheets. Generally, carbons formed at 873 K are still defective and include dangling bonds, which are oxidized upon exposure to air. Hence, Ni_2_-CPD_Py_873(1) contains a small amount of oxygen. At a higher carbonization temperature (973 K), the oxygen amount decreases, indicating a decrease in dangling bonds. This is attributed to further development of the graphene sheets. Notably, the N and Ni contents are well retained up to 973 K. Thus, a hybrid material containing a large fraction of heteroatoms can be produced.

**Table 1:** Elemental compositions of the samples

	Elemental composition (wt%)				
Sample	C	H	N	Ni	O
Ni_2_-CPD_Py_^a^	76.8	3.4	11.7	8.2	0
Ni_2_-CPD_Py_873(1)	75.7	2.0	10.5	9.1	2.7
Ni_2_-CPD_Py_973(1)	78.1	1.1	10.2	9.4	1.2

^a^For Ni_2_-CPD_Py_, the composition is calculated from its molecular formula.

**Fig. 3:**
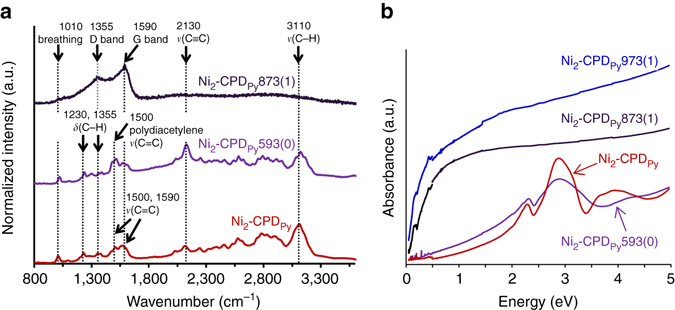
Raman and UV-vis absorption spectra of Ni_2_-CPD_Py_ and heat-treated samples. **a** Raman spectra: peak assignments are based on theoretical calculations (Supplementary Fig. 12) and references^36, 39^. **b** Absorption spectra measured by FT-IR (<0.4959 eV) and UV-vis-NIR spectrometry (>0.4959 eV)

The Ni_2_-CPD_Py_ chemical structure transition was analysed by Raman spectroscopy (Fig. 3a). Some of the major peaks in the Raman spectrum of Ni_2_-CPD_Py_ can be ascribed as follows: breathing, 1010 cm^–1^; *δ*(C–H), 1230 and 1355 cm^–1^; *ν*(C=C), 1500 and 1590 cm^–1^; *ν*(C≡C), 2130 cm^–1^; and *ν*(C–H), 3110 cm^–1^, from the results of simulation using the Gaussian 09 software^35^, (Supplementary Methods, Supplementary Figs. 11 and 12). Most peaks are retained in Ni_2_-CPD_Py_593(0); this is in agreement with the structure change shown in Fig. 2. A peak at 1500 cm^−1^ becomes intense in the polymer, and this reflects the formation of a poly(diacetylene) backbone (Supplementary Fig. 12). In Ni_2_-CPD_Py_873(1), most peaks disappear, and only broad D- and G-bands^36^ originating from intervalley scattering^37–39^ appear, indicating that the well-defined chemical structure of the precursor polymer framework is lost and the resulting OCF consists of defective graphene sheets (Fig. 2l), like zeolite-templated carbons (ZTCs) and ordered mesoporous carbons^2^. Ni_2_-CPD_Py_ absorption spectrum (Fig. 3b) displays Q and Soret bands of the Ni^2+^ porphyrin unit (2.31 and 2.89 eV, respectively). Almost no energy shift is observed in Ni_2_-CPD_Py_593(0), strongly suggesting that the electric structures of these units are retained in Ni_2_-CPD_Py_593(0). This agrees with the structure in Fig. 2h–j. Conversely, Ni_2_-CPD_py_873(1) and Ni_2_-CPD_py_973(1) display a broad absorption band from the mid-IR to UV region assigned to the interband transitions of the graphene sheets. Since the absorption of this transition is too strong, the absorption bands of the Ni^2+^ porphyrin unit are veiled and their presence cannot be confirmed in Fig. 3b. As shown later, the chemical states of Ni in the carbonized samples were analyzed also by X-ray absorption fine structure (XAFS) measurements of the Ni–*K* edge.

**Fig. 4:**
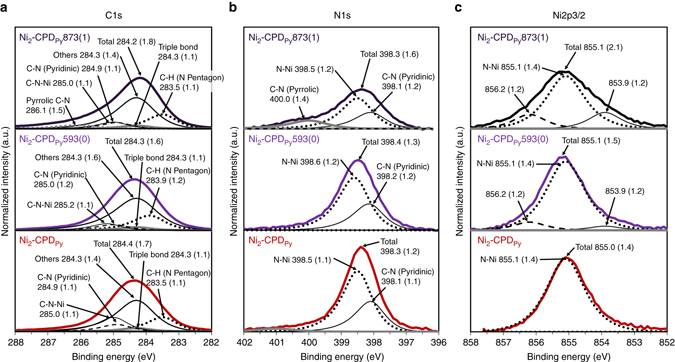
X-ray photoelectron spectroscopy results of Ni_2_-CPD_Py_ and heat-treated samples. **a** C1s, **b** N1s, **c** Ni2p_3/2_. Numbers before parenthesis indicate binding energy in eV. Numbers inside the parenthesis indicate FWHM of spectra. In **a** and **b**, experimentally obtained peaks are deconvoluted into several peaks determined by theoretical calculations (Supplementary Fig. 16). In **c**, FWHM of the N–Ni peak is determined as 1.4 eV from the result of Ni_2_-CPD_Py_, and the broadened parts in the heat-treated samples were deconvoluted into three peaks including two additional peaks that have lower (853.9 eV) and higher (856.2 eV) binding energies than that of N–Ni

The formation of graphene sheets renders the OCFs electrically conductive. At room temperature, Ni_2_-CPD_py_ and Ni_2_-CPD_py_593(0) were highly insulating (resistivity *ρ* > 1 TΩ cm). Meanwhile, in Ni_2_-CPD_py_873(1) and Ni_2_-CPD_py_973(1), a significant decrease in *ρ* (33.8 and 18.6 Ω cm, respectively at room temperature) was observed. Thus, unlike conventional organic-based frameworks, OCFs are equipped with electric conductivity. As the temperature decreased, both samples exhibited an increase in resistivity, indicating that both are semiconductors (Supplementary Fig. 13). The activation energies were estimated as 0.050–0.091 eV for Ni_2_-CPD_py_873(1) and 0.015–0.022 eV for Ni_2_-CPD_py_973(1). The microstructures of OCFs were further studied by X-band electron paramagnetic resonance (EPR) analysis (Supplementary Fig. 14). Ni_2_-CPD_Py_873(1) at 5.0 K displayed an absorption line (*g* ~ 2) with a sharp line-width (2.8 mT). These results indicate the formation of organic radicals derived from dangling bonds generated during the carbonization process. Conversely, Ni_2_-CPD_Py_973(1) exhibited broad absorptions and no signals attributed to radicals were observed. The broad peak is assigned to coupling with anisotropic spins in the six-coordinated paramagnetic Ni^2+^ ions generated during heat treatment. The structural change of Ni_2_-CPD_Py_ was further analysed by X-ray photoelectron spectroscopy (XPS, Fig. 4). No significant changes were observed in the C1s, N1s, and Ni2p_3/2_ XPS spectra during the transition from Ni_2_-CPD_Py_ to Ni_2_-CPD_Py_593(0). This is explained by the change in structure shown in Fig. 2. The C1s spectra of Ni_2_-CPD_Py_ and Ni_2_-CPD_Py_593(0) are well in agreement with results from theoretical calculations (Supplementary Figs. 15 and 16)^35, 40–44^ as well as the positions and full width at half maximum (FWHM) of the deconvolution peaks. Moreover, the C1s, N1s, and Ni2p_3/2_ spectra of Ni_2_-CPD_Py_873(1) indicate that the chemical environment of these atoms differ only slightly from those in Ni_2_-CPD_Py_593(0), despite the disappearance of the well-defined phenyl groups, C≡C, and C–H bonds (Fig. 3a). In Ni_2_-CPD_Py_873(1), the N1s spectrum slightly broadens (change in FWHM) because of the appearance of a small C–N (pyrrolic) component at 400.0 eV. These moieties may be formed by thermal conversion of the pyridyl groups into the carbonaceous framework containing the pyrrolic structure, or by cleavage of porphyrin rings followed by hydrogen addition to the free pyrrolic N. The Ni2p_3/2_ spectrum of Ni_2_-CPD_Py_ broadens after heat treatment (two peak components at 856.2 and 853.9 eV). These components are ascribed to the oxidized Ni species, carbides, or metallic Ni^45^.

### Chemical environment of Ni in the carbon matrix

**Fig. 5:**
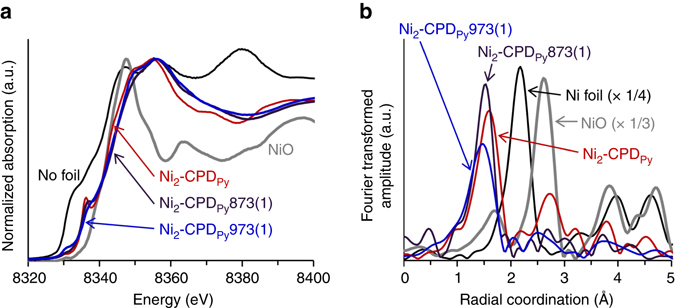
X-ray absorption fine structure results of Ni_2_-CPD_Py_ and its heat-treated samples. **a** XANES spectra. **b** Pseudo-radial structural functions calculated from EXAFS patterns. The data of Ni foil and NiO are also shown for comparison

The XAFS of the Ni–*K* edge was also analysed by synchrotron X-ray absorption spectroscopy [X-ray absorption near edge structure (XANES) spectra in Fig. 5a]. The Ni–*K* edge energy (~ 8333 eV) of Ni_2_-CPD_Py_ lies between those of Ni foil (8329 eV) and NiO (8336 eV). This reflects the intermediate oxidation state of Ni in Ni_2_-CPD_Py_. The Ni_2_-CPD_Py_ XANES spectrum exhibits a characteristic peak at 8336 eV, corresponding to the 1 s to 4p_z_ transition^46^. This is typical of a planar porphyrin^47^ and phthalocyanine^46^ where the Ni coordinates with four nitrogen atoms in the Ni–N_4_ unit. Notably, the Ni_2_-CPD_Py_873(1) XANES spectrum is almost unchanged from that of Ni_2_-CPD_Py_, indicating the retention of the Ni–N_4_ unit even after carbonization. In Ni_2_-CPD_Py_973(1), a shoulder appears at 8329 eV, suggesting the formation of a small amount of metallic Ni due to the partial decomposition of the porphyrin moieties. However, the overall spectrum is still well retained.

The pseudo-radial structure functions were next calculated using the Ni–*K* edge extended X-ray absorption fine structure (EXAFS) spectra for Ni_2_-CPD_Py_ and its carbonized derivatives, Ni foil, and NiO (Fig. 5b). Ni_2_-CPD_Py_ exhibits an intense peak at 1.56 Å, corresponding to the four N atoms coordinated to the Ni atom. The carbonized samples display very similar patterns to that of Ni_2_-CPD_Py_, confirming the retention of the Ni–N_4_ unit after carbonization. The precise distance between Ni and N and its coordination number were calculated with FEFF8.2 (Supplementary Table 1). Ni_2_-CPD_Py_873(1) and Ni_2_-CPD_Py_973(1) retained the coordination numbers 3.8 and 3.4, respectively. This is close to the initial number (4).

The chemical environment of the Ni^2+^ ions was studied also by magnetic susceptibility analysis (Supplementary Fig. 17). Since Ni^2+^ ions in the square planar coordination geometry are diamagnetic, Ni_2_-CPD_Py_ and Ni_2_-CPD_Py_593(0) seldom respond to an external magnetic field. Their minute responses are ascribed to minor impurities. Ni_2_-CPD_Py_873(1) retains a weak magnetization, indicating that the square planar coordination is well retained in the sample; this is in agreement with XAFS data. These samples almost obey the Curie-Weiss law in the range of 5.0–300 K, with small Curie constants (*C* = 6.3 × 10^−6^, 15 × 10^−6^ and 87 × 10^−6^ cm^3^ g^−1^ K, respectively). Conversely, Ni_2_-CPD_Py_973(1) data deviate from this law. This is attributed to the temperature-independent paramagnetic term, *χ*_*p*_, corresponding to Pauli’s paramagnetism of the graphene sheets and Ni metal cluster and agrees with results from XANES (Fig. 5a; curve fitting: *χ*_*p*_ = 13.7 × 10^−6^ cm^3^ g^−1^ and *C* = 202 × 10^×6^ cm^3^ g^−1^ K). The increase in *C* is attributed to the generation of six-coordinated paramagnetic Ni^2+^ ions formed by heat treatment. If all the Ni^2+^ ions are converted into six-coordinated species, then *C* = 1391 × 10^−6^ cm^3^ g^−1^ K (*S* = 1, *g* = 2.0). Since the value of Ni_2_-CPD_Py_973(1) is 14.5% of this assumption, 85.5% of Ni^2+^ ions are expected to retain the square-planar coordination geometry, even after heat treatment at 973 K.

### Electrochemical catalysis

**Fig. 6:**
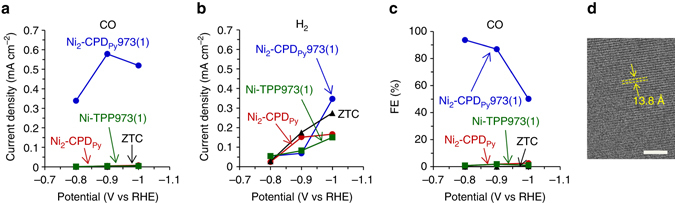
Examination of selective CO_2_ electro-reduction into CO. **a**, **b** Partial current densities used for (**a**) CO and (**b**) H_2_ generation on the samples in CO_2_-saturated 0.1 M KHCO_3_. **c** FE for CO generation. **d** TEM image of a reference ordered microporous carbon, ZTC

Compared to carbon materials, organic-based frameworks have a great advantage of chemical designability. In the latter materials, specific molecular blocks can be integrated three-dimensionally with structure order, by which a variety of unique functions can be achieved. However, they are intrinsically not electrically conductive unlike carbon, and are necessarily deposited as thin films on good conductors for electrochemical applications^48–50^. The proposed OCFs are expected to provide new electrocatalyst designs in which active sites are embedded in electrically conductive frameworks with structure regularity. To prove this concept, we have examined the electrocatalytic activity of the porphyrin center (Ni–N_4_ unit) retained in Ni_2_-CPD_Py_973(1). Unlike the cases of Fe and Co, Ni-based complexes are generally poor in electrocatalysis for oxygen-reduction reaction. However, specific Ni cyclam complexes^51–54^ or Ni-N-modified graphene^55^, which have Ni–N_4_ sites, have been reported to show unique electrocatalysis for the CO_2_ reduction into CO without significant H_2_ evolution and with a high Faradaic efficiency (FE) of ca. 90%. Additionally, this selective CO_2_ reduction has rarely been reported in carbonaceous Ni/N/C materials made from Ni-based complexes. Figure 6 summarizes the comparison of selective CO_2_ reduction activities of Ni_2_-CPD_Py_973(1) and three reference materials, characterized by the method reported elsewhere^55^. Ni_2_-CPD_Py_973(1) shows apparent CO_2_ reduction catalysis into CO (Fig. 6a) even without any conductive additives, while the activity for H_2_ evolution is quite low below −0.9 V vs. RHE (reversible hydrogen electrode) (Fig. 6b). Thus, Ni_2_-CPD_Py_973(1) achieves high FE of 94 and 87% at −0.8 and −0.9 V, respectively (Fig. 6c). To further investigate the uniqueness of the OCF catalysis, the same measurement was applied to three reference materials: ZTC, Ni_2_-CPD_Py_, and Ni-TPP carbonized at 973 K for 1 h [Ni-TPP973(1)]. ZTC is an existing ordered microporous carbon prepared by using zeolite as a hard template^2^, and has the structure periodicity of 13.8 Å (Fig. 6d), which is close to that of Ni_2_-CPD_Py_973(1). ZTC has an electrically conductive framework which comprises mainly of *sp* ^2^ carbons^56^, but it does not possess catalysis sites including metal species. ZTC shows no catalysis in Fig. 6a, and this result clearly indicates that the ordered carbonaceous framework itself can never reduce CO_2_. Ni_2_-CPD_Py_ also shows no catalysis in Fig. 6a despite the presence of the Ni-N_4_ unit, because the organic crystal of Ni_2_-CPD_Py_ is not electrically conductive. Next, we discuss the active site. In the cases of Fe/N/C and Co/N/C electrocatalysts for oxygen reduction reaction, catalysis sites often exist as disordered forms consisting of metal species, N, and C. Hence, it is necessary to examine the catalytic activity of disordered Ni/N/C structure towards the selective CO_2_ reduction into CO. For this purpose, Ni-TPP973(1) was prepared as a representative Ni/N/C material by the same carbonization procedure as that for Ni_2_-CPD_Py_973(1), from a common Ni-based porphyrin Ni-TPP. During the heat treatment, Ni-TPP is decomposed involving cleavage of Ni–N bonds, and it turns into the mixture of a disordered Ni/N/C framework and Ni metal aggregation (Supplementary Fig. 18). The resulting Ni-TPP973(1) shows no catalysis in Fig. 6a, indicating that the disordered Ni/N/C structure is not active, and the Ni–N_4_ unit is the active site in Ni_2_-CPD_Py_973(1). Figure 6 thus proves that the selective CO_2_ reduction catalysis of Ni_2_-CPD_Py_973(1) can be achieved by the intermediate structure of organic-based frameworks and carbon materials, in which molecular catalysis sites (Ni–N_4_) are embedded in the conductive framework. As mentioned above, conventional M/N/C catalysts have disordered structures and this has hampered the basic understanding between the structure and catalysis. With its certainly determined catalysis sites, Ni_2_-CPD_Py_973(1) can be a good platform to investigate the fundamentals of carbonaceous electrocatalysts.

The advantage of OCFs compared to conventional carbon materials is chemistry-based better controllability. Though the present OCFs are poorly porous (Supplementary Table 2), it is possibly improved by introducing volatile groups at designed sites of the starting molecules. Replacing Ni with other metals such as Fe, Co, Cu, Pt, and Pd can also widen the versatility of OCFs. Moreover, the exterior shape of the crystal can be also controlled based on the existing methods, for example a reprecipitation method^57–60^ (Supplementary Fig. 19) to achieve additional function^59^. By their chemical designability, OCFs are expected to be further developed hereafter.

## Discussion

In summary, the direct conversion of organic crystal into OCFs was demonstrated. The successive thermal conversion process actually comprises two steps. A molecular crystal of Ni_2_-CPD_Py_ is first thermally converted into a crystalline covalent-organic framework, and is further converted into OCFs that inherit the periodic structure and the Ni–N_4_ unit in the precursor organic crystal. The successful conversion is due to the following properties of Ni_2_-CPD_Py_. First, absence of volatile moieties like paraffin structures, oxygen, halogens, and sulphur. Second, presence of well-arrayed diacetylene moieties that can be thermally crosslinked to form a heat-stable polymer. Third, the presence of thermally stable (~ 973 K) organic moiety (metal porphyrin unit). On the basis of this strategy, a variety of OCFs could be synthesized, probably also from organic molecules other than Ni_2_-CPD_Py_. This new pathway allows the preparation of OCFs with molecularly controlled chemical structures that can be considered fusion materials of organic-based frameworks and carbon materials.

## Methods

### Materials

Ni_2_-CPD_Py_ and H_4_-CPD_Py_ were synthesized according to literature^22, 25^. Heat treatment of Ni_2_-CPD_Py_ and H_4_-CPD_Py_ was performed at a heating rate of 5 K min^−1^ ramped at a designed temperature (593, 873, or 973 K, N_2_ flow) by using a tubular furnace. In the case of 593 K, heating was stopped immediately when the temperature reached 593 K. The samples thus obtained are Ni_2_-CPD_Py_593(0) and H_4_-CPD_Py_593(0). The sample name is expressed as follows: M-CPD_Py_X(Y) for M=Ni_2_ or H_4_, X is the treated temperature (K), and Y is the period of the treatment (h). In the case of 873 and 973 K, the temperature was maintained at the target temperatures for 1 h. As a reference, 5, 10, 15, 20-tetraphenyl-21H,23H-porphine nickel(II) (≥95%, Sigma-Aldrich) was carbonized at 973 K for 1 h by the same manner as that for Ni_2_-CPD_Py_973(1). The sample thus obtained is Ni-TPP973(1). Zeolite-templated carbon was synthesized by the method reported elsewhere^61^.

### Characterization

Porphyrin TG curves were measured by a Shimazu TGA-51 thermogravimetric analyser (N_2_ flow, ≤1073 K, H_4_-CPD_Py_/Ni_2_-CPD_Py_). TPD patterns were measured using a Rigaku ThermoMass Photo spectrometer (10 K min^−1^, ≤1073 K, He flow). The afforded residue was observed by TEM (JEM-2010/JEM-2200FS, JEOL). DSC curves were recorded on a Mettler DSC1 STARe (≤623 K, 10 K min^−1^, N_2_ flow). To confirm the presence of the monomer/oligomers in Ni_2_-CPD_Py_593(0), the sample was dispersed in chloroform and analysed by ^1^H NMR (Bruker Avance III 400) and MALDI-TOF-MS (Bruker Autoflex Speed). ^13^C CP-MAS NMR spectra were measured on a JEOL JNM-ECA800 (800 MHz) spectrometer. The sample was packed into a 2.5-mm zirconia rotor, and the measurement was carried out using two-pulse phase-modulated decoupling (30 kHz, 1 s recycle delay, 3 ms contact time, π/2 pulse width = 2.43 μs at 69.5 W, and 2048 scans). The CH_2_ peak of the external adamatane standard was 29.5 ppm. Spectra were processed with Delta NMR Software (v 5.0) using conventional techniques (100 Hz line broadening window function). The position of Ni in Ni_2_-CPD_Py_873(1) was analysed by the HAADF-STEM technique (Titan^3^ G2 60–300 Double Cs-Corrector, FEI Company; 300 V). Elemental analysis of the carbonized samples was carried out with a Yanaco JM10 analyser. The sample underwent combustion (flow = 20%-O_2_ + 80%-He); gasses generated were converted into CO_2_, H_2_O, and N_2_ to determine the respective C, H, and N amounts; Ni remained as oxidized ash. The Ni content was determined by assuming its composition as NiO; the amount of O was calculated by subtracting the amount of C, H, N, and Ni from the initial sample weight. Raman spectra were measured with a Jasco NRS-3100 (532.2 nm line). Absorption spectra were measured from samples moulded into KBr pellets (Nicolet 6700 FT-IR spectrometer, 0.04949–0.4959 eV/Perkin-Elmer Lambda750A UV-vis-NIR spectrometer, 0.4959–4.959 eV). The temperature-dependent electric resistance was measured with a two-electrode method for high resistance samples [Ni_2_-CPD_Py_, Ni_2_-CPD_Py_593(0), Ni_2_-CPD_Py_873(1); pellet diameter = 3 mm] and a four-probe method for low resistance Ni_2_-CPD_Py_973(1) (rod-shape: 3.0 × 0.68 × 0.37 mm). Ni_2_-CPD_Py_873(1) and Ni_2_-CPD_Py_973(1) were mixed with 5 wt% binder polymer (PTFE). The sample was placed in a Sumitomo SRDK-101D cryogenic refrigerating system. Electric contacts were prepared using Tokuriki #8560 gold paste and 25 μm gold wires. In the two-probe method, force-voltage current measurements were performed using a Keithley 6517 A electrometer. In the four-probe method, a constant current (0–2 μA) was applied (Advantest R6161). The voltage was measured by a Hewlett-Packard 3458 A digital multimeter. XPS spectra were measured with a JEOL JPS-9200. To avoid charge build-up, a solution of Ni_2_-CPD_Py_ in chloroform was spin-coated on an Al substrate (purity = 99.999, Al-Kα radiation, spot size = 3 mm). The substrate was heat treated (≤593 K) to prepare Ni_2_-CPD_Py_593(0) and its XPS spectra were recorded. Subsequently, the substrate was heat treated (873 K, 1 h) to prepare Ni_2_-CPD_Py_873(1) and its XPS spectra were recorded. Ni–*K* edge XAFS measurements, before and after carbonization of Ni_2_-CPD_Py_, were performed in transmission mode (in air, room temperature, synchrotron radiation BL14B2 beam line, SPring-8). The recorded spectra were normalized and fitted by REX2000 (Rigaku). The precise Ni to N distance and coordination number were calculated from the EXAFS results by using FEFF8.2. Magnetic susceptibility data were collected in the temperature range of 5.0–300 K in an applied field of 10 kG using a Quantum Design MPMS2 SQUID magnetometer. X-band EPR data were recorded on a JEOL JES-FA100 spectrometer equipped with an Oxford ESR900 continuous-flow liquid He cryostat. N_2_ and CO_2_ adsorption isotherms were measured at 77 K and 298 K, respectively (MicrotracBEL Corp. BELMAX). In the N_2_ adsorption isotherm, the specific surface area was calculated by the Brunauer–Emmett–Teller (BET) method in the pressure range of *P*/*P*_0_ = 0.05–0.35, and the total pore volume (*V*_N2_) was calculated at *P*/*P*_0_ = 0.96. In the CO_2_ adsorption isotherm, the pore volume (*V*_CO2_) was calculated by the Dubinin–Radushkevich equation.

### Data availability

Crystallographic data (CIF files) for Ni_2_-CPD_Py_ and Ni_2_-CPD_Py_593(0) have been deposited with the Cambridge Crystallographic Data Centre as supplementary publications. CCDC 1552441 (Ni_2_-CPD_Py_) and CCDC 1552442 (Ni_2_-CPD_Py_593(0)) contain the supplementary crystallographic data. These data can be obtained free of charge from the Cambridge Crystallographic Data Centre via www.ccdc.cam.ac.uk/data_request/cif. PXRD analysis, construction of a model structure of Ni2-CPDPy873(1), computational simulations, and CO2 reduction measurements are provided in Supplementary Methods. All other data supporting the findings of this study are available within the article and its Supplementary Information.

## Electronic Supplementary Material

Supplementary Information

Supplementary Movie 1

Supplementary Movie 2

Supplementary Movie 3_2_-CPD_Py_873(1) structure shown in Fig. 2l-(B)

Peer Review File
