# Supplementary material for: Synthesis of ordered carbonaceous frameworks from organic crystals
Source: Nat Commun. 2017 Jul 24;8:109. doi: 10.1038/s41467-017-00152-z (PMC5524644; doi:10.1038/s41467-017-00152-z)
Supplement: Supplementary file 1 — Supplementary Information [file 41467_2017_152_MOESM1_ESM.pdf]

File Name: Supplementary Information
Description: Supplementary Figures, Supplementary Tables and Supplementary Methods

File Name: Supplementary Movie 1
Description: 3D Rotation of the Ni_2-CPD_{Py} structure shown in Fig. 2f.

File Name: Supplementary Movie 2
Description: 3D Rotation of the Ni_2-CPD_{Py}593(0) structure shown in Fig. 2j.

File Name: Supplementary Movie 3
Description: 3D Rotation of the Ni_2-CPD_{Py}873(1) structure shown in Fig. 2l-(B).

File Name: Peer Review File
Description:

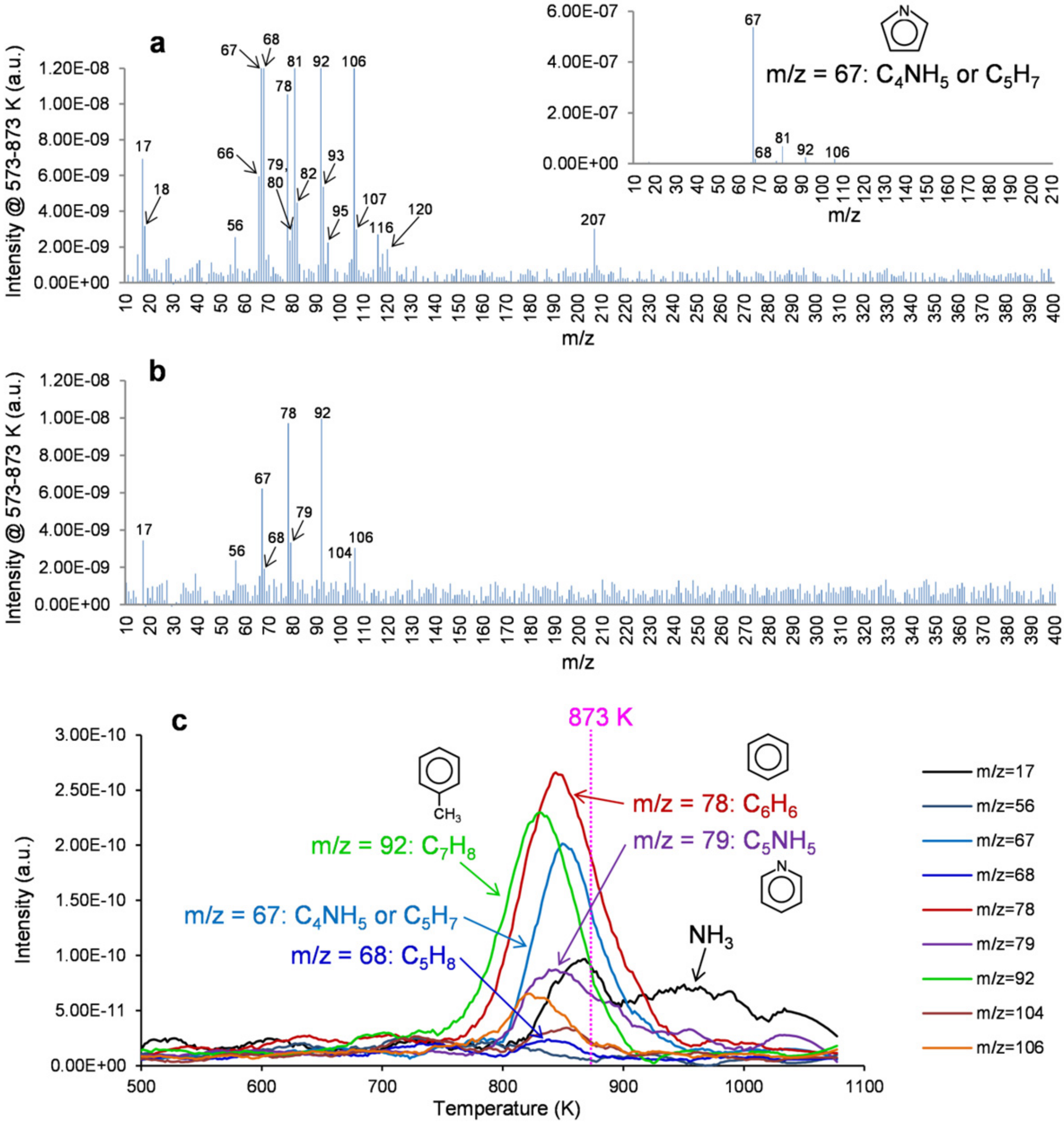

Supplementary Figure 1. The results of temperature-programmed desorption (TPD) measured on a thermogravimetry/photoionization mass spectroscopy (TG-PI-MS) instrument. **a**,**b**, PI-MS spectra integrated in 573-873 K for H_4-CPD_{Py} (**a**) and Ni_2-CPD_{Py} (**b**). Since H_4-CPD_{Py} shows a very intense peak of m/z = 67, an enlarged spectrum is shown in (**a**), and its overall spectrum is shown as an inset. The m/z = 67 peak is much weakened in Ni_2-CPD_{Py}, and therefore, it is ascribed mainly to C_4NH_5. Thermal stability of free-base porphyrin is much lower than that of Ni-porphyrin, so the free-base porphyrin moieties in H_4-CPD_{Py} could be decomposed to release a large amount of C_4NH_5. At the same time, C_5H_7 species could be formed by a complex

decomposition reaction occuring in H_4-CPD_{Py}. **c**, TPD patterns of major species in Ni_2-CPD_{Py}. m/z = 78, 79, and 92 are ascribed to C_6H_6, C_5NH_5, and C_7H_8, respectively. C_5NH_5 could be derived from pyridyl groups, while C_6H_6 and C_7H_8 could be derived from phenyl moieties in Ni_2-CPD_{Py}. Unlike the case of H_4-CPD_{Py}, m/z = 67 cannot be simply ascribed to C_4NH_5, because most of the characterization data on Ni_2-CPD_{Py}873(1) suggest that the Ni-porphyrin moieties are rarely decomposed. This is thus also ascribed to C_5H_7 which could be formed during the thermal decomposition of phenyl moieties and/or polydiacethylen backbones.

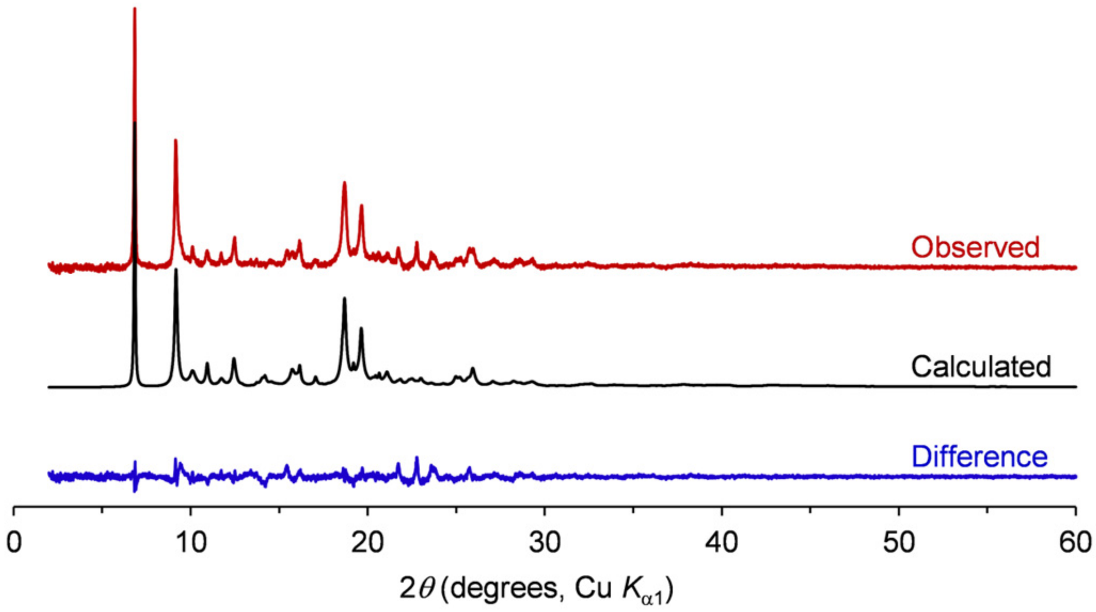

Supplementary Figure 2. PXRD pattern and the profile fitting results of Ni_2-CPD_{Py}. Crystallographic parameters, Formula: $C_{92}H_{48}N_{12}Ni_2$, M: 1438.83, Crystal system: monoclinic, Space group: $P2_1/c$, a: 13.040(10) Å, b: 25.897(14) Å, c: 10.824(8) Å, β: 105.37(3)°, V: 3524(4) $Å^3$, Z: 2, T: 298 K, D_{calc}: 1.36 g cm^{-3}, R_{wp}: 2.36%, S: 1.78. Crystallographic data (CIF file) have been deposited with the Cambridge Crystallographic Data Centre as supplementary publications (CCDC 1552441).

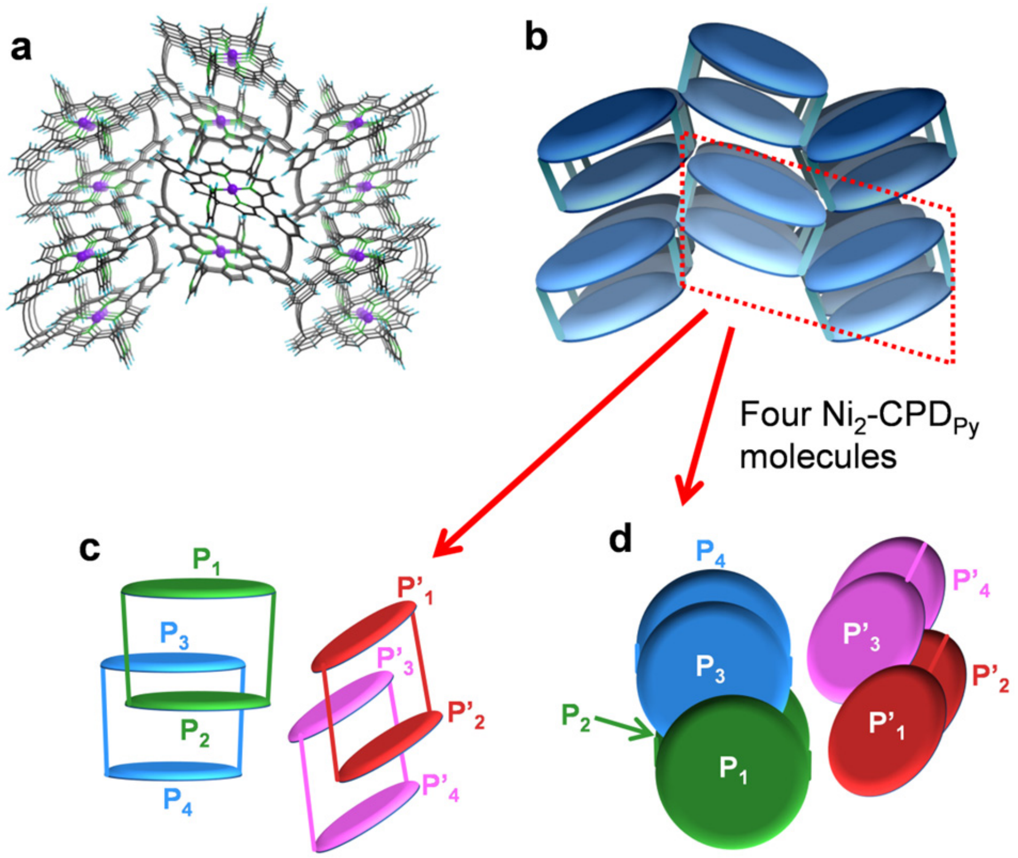

Supplementary Figure 3. The detail illustration for the structure of Ni_2-CPD$_{Py}$. a, A perspective image of Ni_2-CPD$_{Py}$ corresponding to Fig. 2f. **b**, A simplified model corresponding to (**a**). A porphyrin and a diacetylene moieties are represented by a disk and a line, respectively. **c**,**d**, Four simplified molecular models viewed from different directions. Each Ni_2-CPD$_{Py}$ molecule is displayed by different colours, and each of porphyrin units is marked with the symbols of P_n (n = 1–4). The porphyrins in the next column are described with the symbols of P_n' (n = 1–4). A movie showing the rotating 3D structure of Ni_2-CPD$_{Py}$ is provided as Supplementary Movie 1.

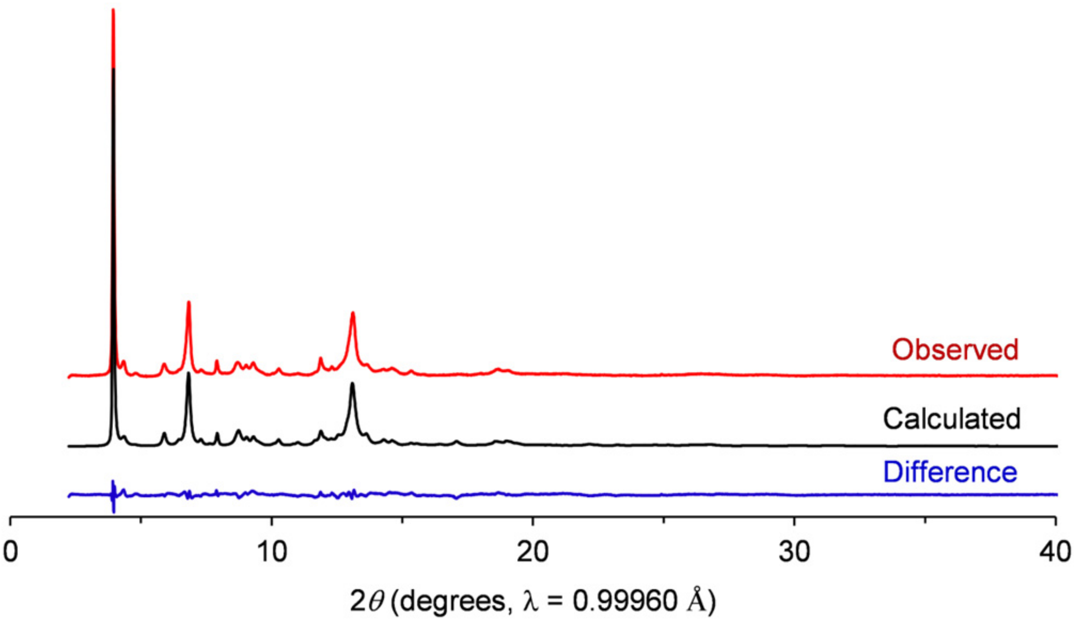

Supplementary Figure 4. PXRD pattern and the profile fitting results of Ni_2-CPD_{Py}593(0). Crystallographic parameters, Formula: $C_{92}H_{48}N_{12}Ni_2$, M: 1438.83, Crystal system: monoclinic, Space group: $P2_1/c$, a: 13.6734(17) Å, b: 29.117(5) Å, c: 9.7438(18) Å, β: 105.232(17)°, V: 3743.1(11) $Å^3$, Z: 2, T: 298 K, D_{calc}: 1.28 g cm^{-3}, R_{wp}: 2.53%, S: 1.45. Crystallographic data (CIF file) have been deposited with the Cambridge Crystallographic Data Centre as supplementary publications (CCDC 1552442).

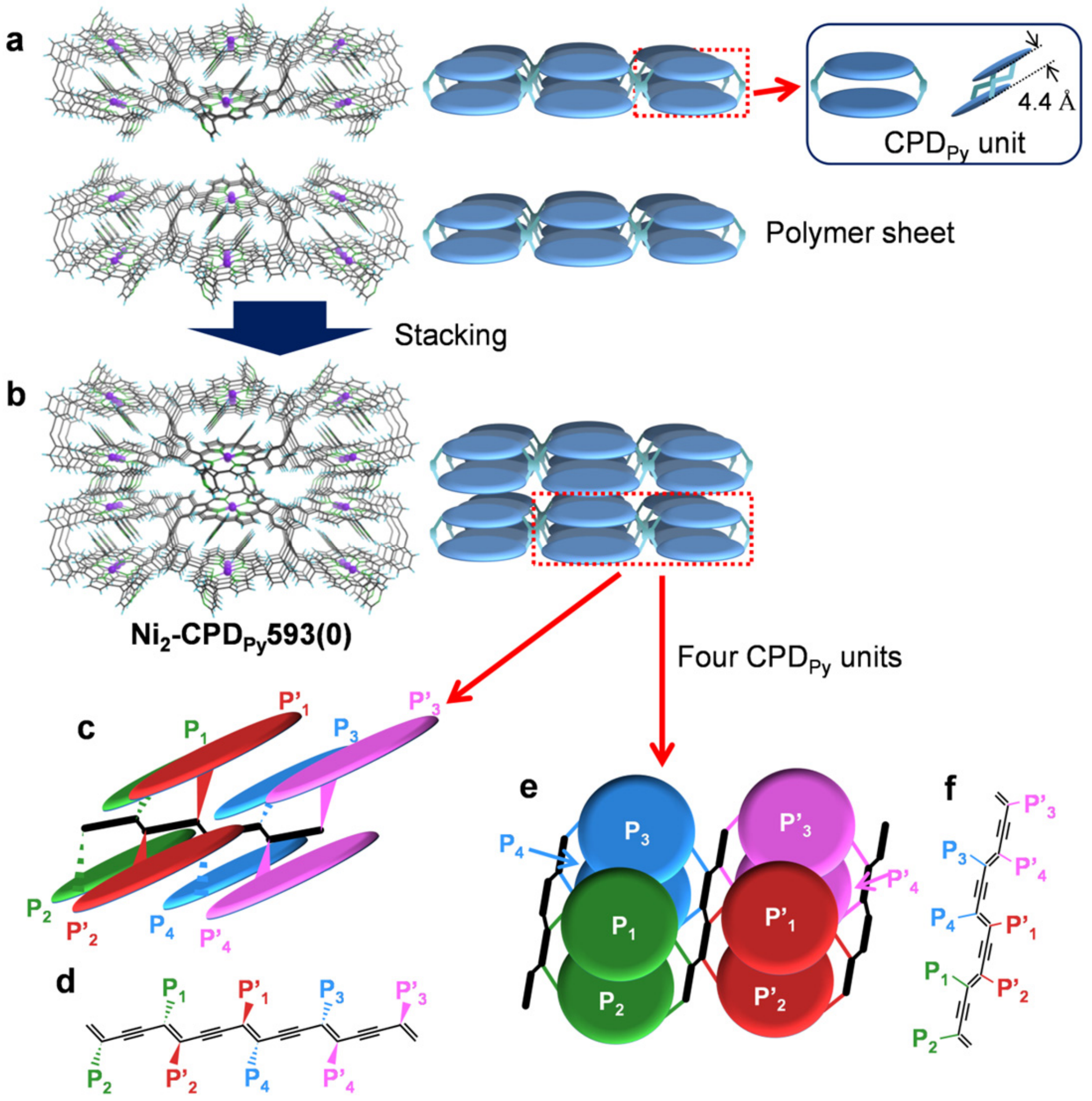

Supplementary Figure 5. The detail illustration for the structure of Ni$_2$-CPD$_{Py}$593(0). a, Individual polymer sheets in Ni$_2$-CPD$_{Py}$593(0). A molecular structure model (i) and a simplified model (ii) are shown. In the simplified model, a porphyrin and a diacetylene moieties are represented by a disk and a line, respectively, as shown in (iii). An inset shows one CPD$_{Py}$ unit viewed from two different angles. **b**, A perspective image of Ni$_2$-CPD$_{Py}$593(0) corresponding to Fig. 2j. **c**, Four CPD$_{Py}$ units viewed from the <010> direction. **d**, A structural diagram corresponding to the top image of Fig. 2h. In (**c**) and (**d**), each of porphyrin units in the same column along the *c*-axis is marked with the symbols of P_n (n = 1–4). The porphyrins in the next column are described with the symbols of P_n' (n = 1–4). The pairs of porphyrins corresponding to the CPD$_{Py}$ unit are displayed by different colours. **e**, Four CPD$_{Py}$ units viewed from the direction of perpendicular to the polymer sheet. (**e**) and (**f**) correspond to (**c**) and (**d**), respectively. In (**c-f**), the poly(diacetylene) backbone is

shown by black colour. A movie showing the rotating 3D structure of Ni_2-$CPD_{Py}593(0)$ is provided as Supplementary Movie 2.

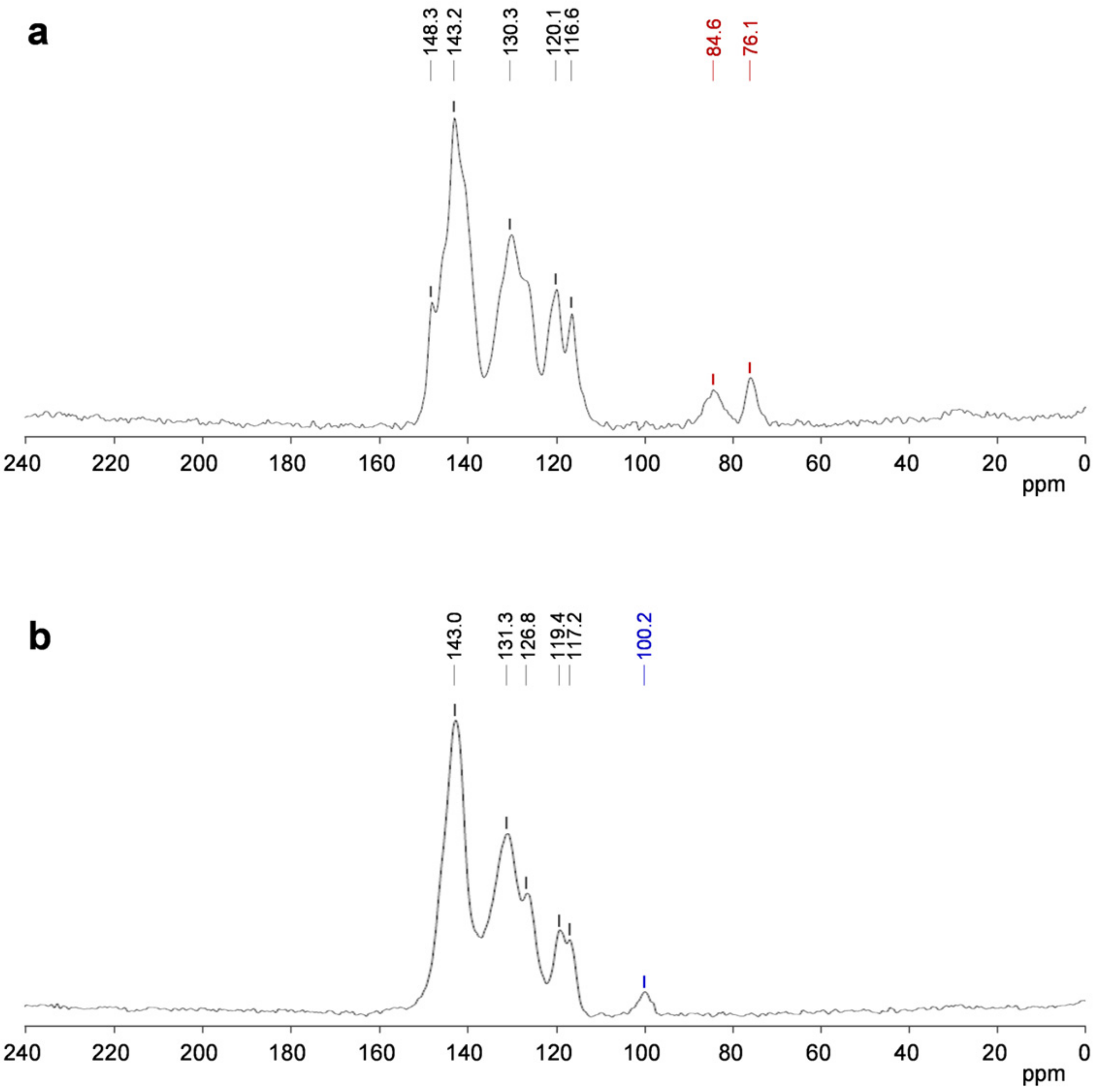

Supplementary Figure 6. **^{13}C CP-MAS NMR spectra. a**, Ni_2-CPD_{Py}. **b**, Ni_2-CPD_{Py}593(0). In (**a**), two peaks at 76.1 and 84.6 ppm are assigned to diacetylenic carbons[26, 32]. Other peaks are derived from aromatic carbons in phenyl groups, pyrydyl groups, and porphyrin frameworks. In its polymerized form (**b**), the peaks of diacetylenic carbons completely disappear and a new peak is found at 100.2 ppm, which is assigned to an acetylene group in a poly(diacetylene) backbone[26]. Thus, the ^{13}C CP-MAS NMR data indicate that diacetyelene chain in Ni_2-CPD_{Py} is completely polymerized into poly(diacetylene) form.

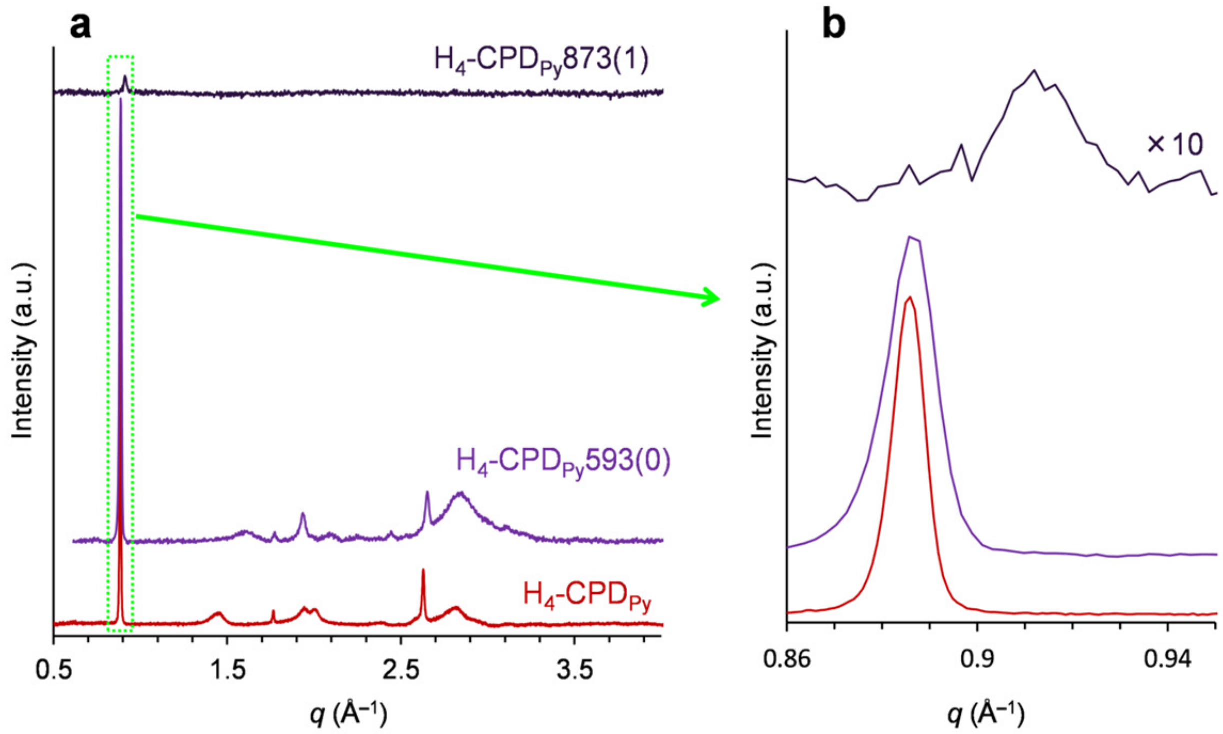

Supplementary Figure 7. PXRD patterns of H_4-CPD$_{Py}$ and its heat-treated samples up to 873 K. a, Overal patterns. **b**, Enlarged patterns of low-angel region. In (**a**), H_4-CPD$_{Py}$ has only several sharp peaks together with some broad peaks, indicating that its solid is not highly crystalline, but contains irregular packing structures. This causes lower polymerization degree than the case of Ni_2-CPD$_{Py}$, as is indicated by its DSC curve (Fig. 1b). The PXRD pattern of the polymerized sample, H_4-CPD$_{Py}$593(0), slightly differs from that of H_4-CPD$_{Py}$, and the resulting polymer seems to still contain irregular packing structures from the presence of several broad peaks. In the carbonized sample at 873 K [H_4-CPD$_{Py}$873(1)], only a very samll peak is detected, as enlarged in (**b**). Thus, the ordered structure of its precursor polymer is almost lost in H_4-CPD$_{Py}$873(1), unlike the case of Ni_2-CPD$_{Py}$873(1).

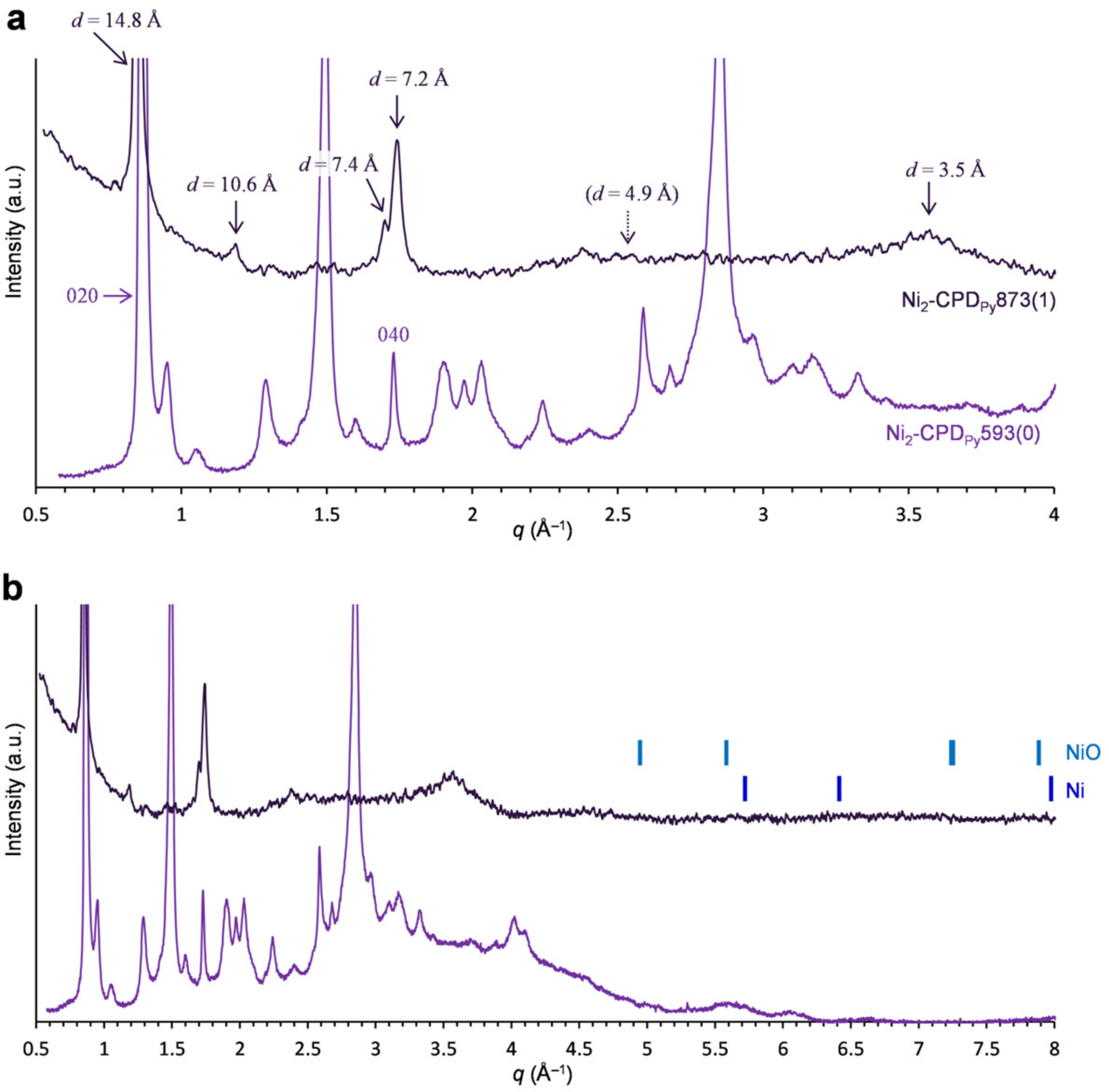

Supplementary Figure 8. Synchrotron PXRD patterns of Ni_2-CPD$_{Py}$593(0) and Ni_2-CPD$_{Py}$873(1). **a**, Low-angle region. For Ni_2-CPD$_{Py}$873(1), *d*-spacings of some peaks are described. **b**, Overall range together with peak positions of Ni (ICDD: 010713740) and NiO (ICDD: 010716719). The acquisition times are 5 and 60 minutes for Ni_2-CPD$_{Py}$873(1) and Ni_2-CPD$_{Py}$593(0), respectively. A major peak (d = 14.8 Å) of Ni_2-CPD$_{Py}$873(1) is derived from the (020) plane of Ni_2-CPD$_{Py}$593(0), and this regularity is clearly observed in Fig. 2m, Supplementary Fig. 9a and b. Its secondary diffraction (d = 7.4 Å, Supplementary Fig. 9c) is also observed, while the tertiary diffraction (d = 4.9 Å, Supplementary Fig. 9c) is very weak. A broad and very weak peak corresponding to d = 3.5 Å is ascribed to the stacking of small graphene sheets, and this is generally

observed in low-crystalline carbonaceous materials. Since its intensity is very weak, only a very small amount of stacking structure is included in Ni_2-CPD_{Py}873(1). The peaks corresponding to d = 7.2 and 10.6 Å cannot be assigned at this moment, but they would be derived from diffraction planes other than (020) and its higher-order planes.

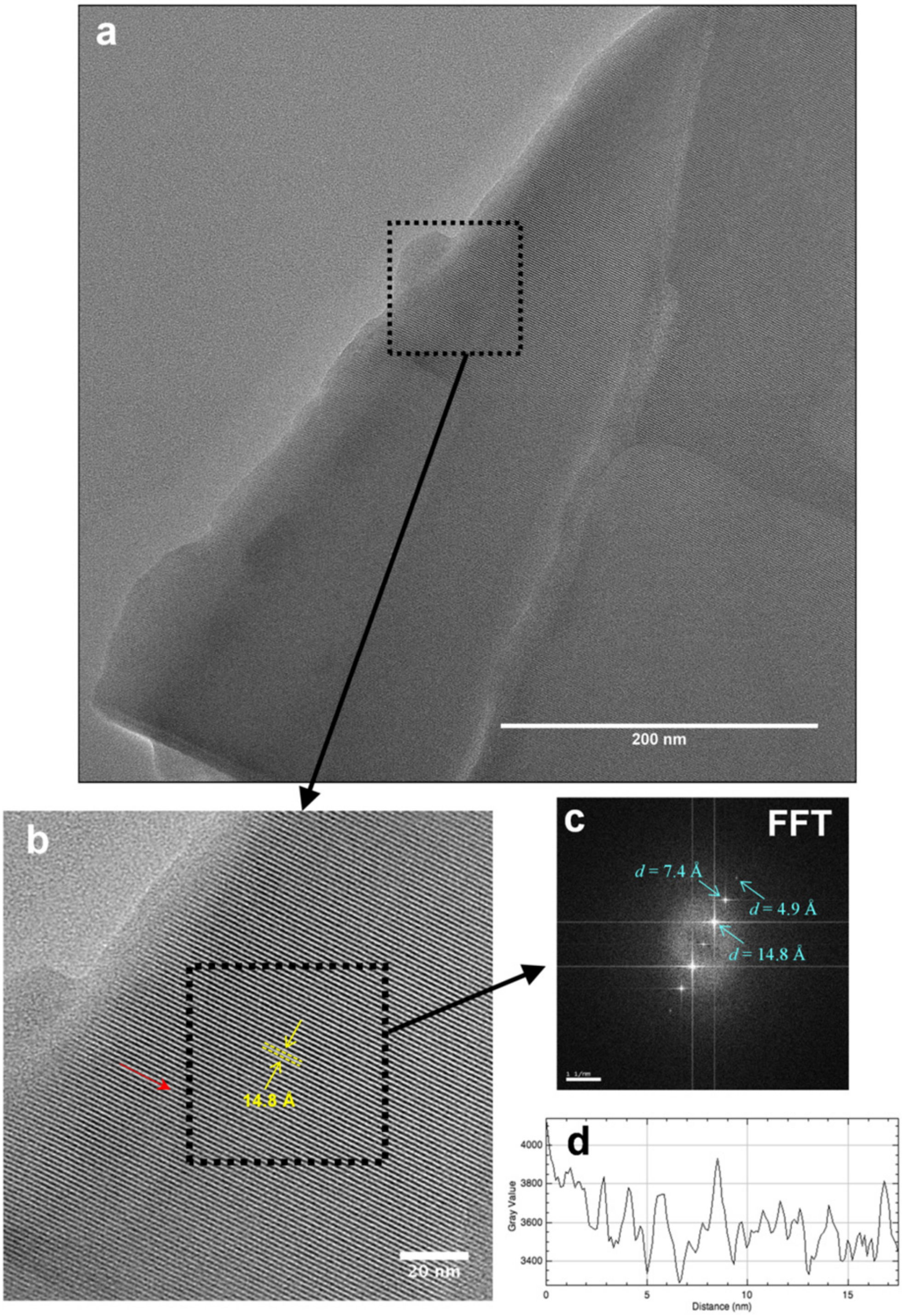

Supplementary Figure 9. TEM analysis results of Ni_2-CPD_{Py}873(1). a, A wide-range TEM image. **b**, An enlarged TEM image corresponding to a square area in (**a**). **c**, A fast Fourier transform (FFT) image corresponding to a square area in (**b**). The *d*-spacing calculated from this FFT image agrees well with the periodicity of the lamella pattern: 14.8 Å and its secondary (7.4 Å) and tertiary (4.9 Å) diffractions. **d**, Brightness profile along a red arrow in (**b**). The arrow is on a black line, corresponding to the diffraction layer containing Ni. The profile shows no periodicity, indicating that Ni is randomly distributed in the diffraction layer.

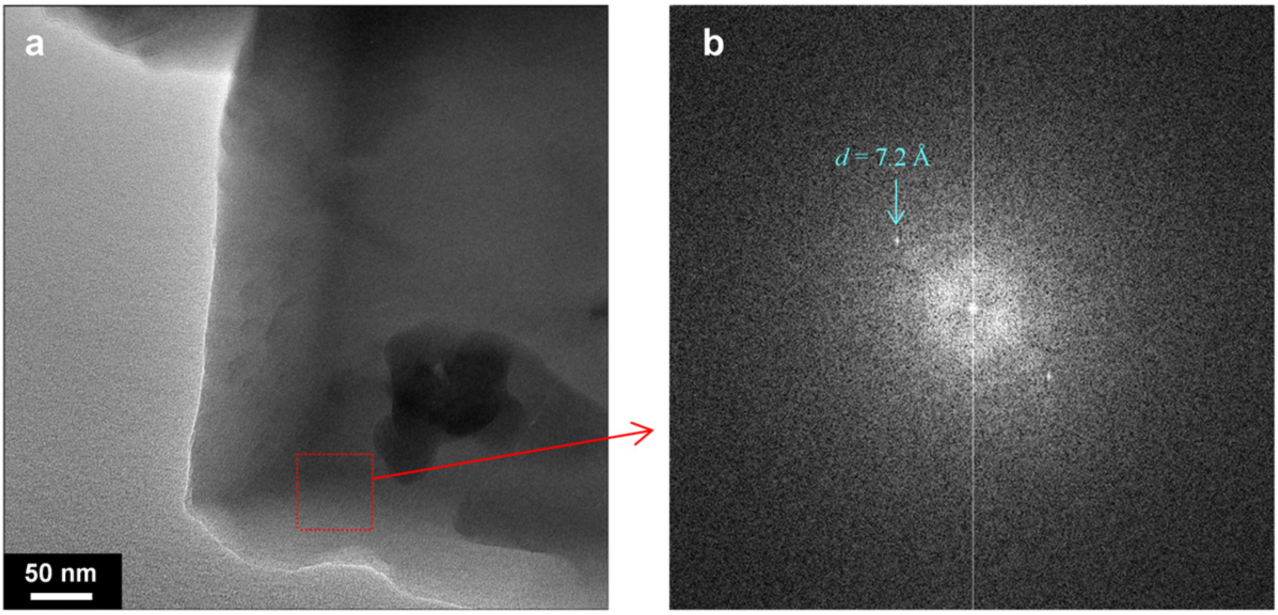

Supplementary Figure 10. TEM analysis of Ni_2-CPD_{Py}873(1) at an angle which excludes the (020) plane. a, A TEM image. The viewing angle was adjusted not to show the (020) plane but to show a diffraction pattern corresponding to d = 7.2 Å. **b**, A FFT image of a square area in (**a**). The diffraction pattern corresponding to d = 7.2 Å is observed. Since there is no (020) plane in this perspective, this is not a higher-order plane of (020).

(a) Partial structure of Ni_2-CPD_{Py}

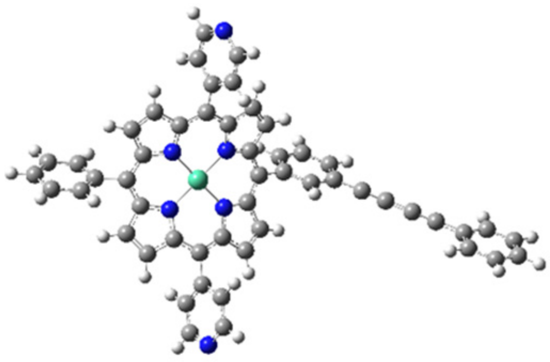

(b) Diacetylene moiety in Ni_2-CPD_{Py}

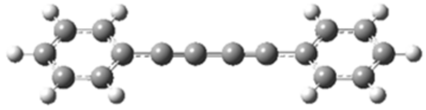

(c) Poly(diacetylene) in Ni_2-CPD_{Py}593(0)

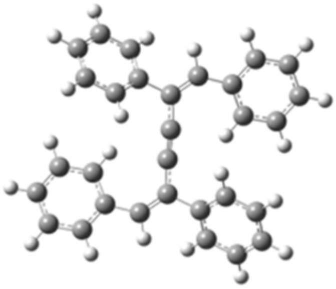

(d) Partial structure of Ni_2-CPD_{Py}593(0)

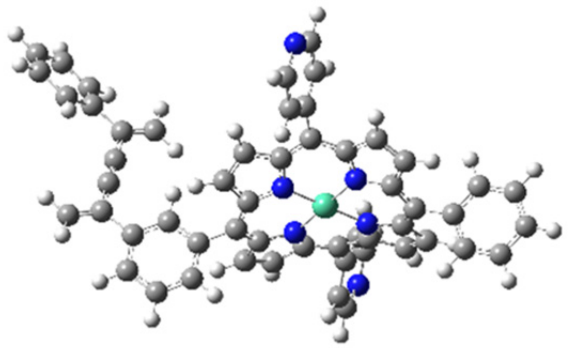

Supplementary Figure 11. Structures used for calculating Raman spectra. a, Partial structure of Ni_2-CPD_{Py}. **b**, Diacetylene moiety. **c**, Poly(diacetylene) in Ni_2-CPD_{Py}593(0). **d**, Partial structure of Ni_2-CPD_{Py}593(0).

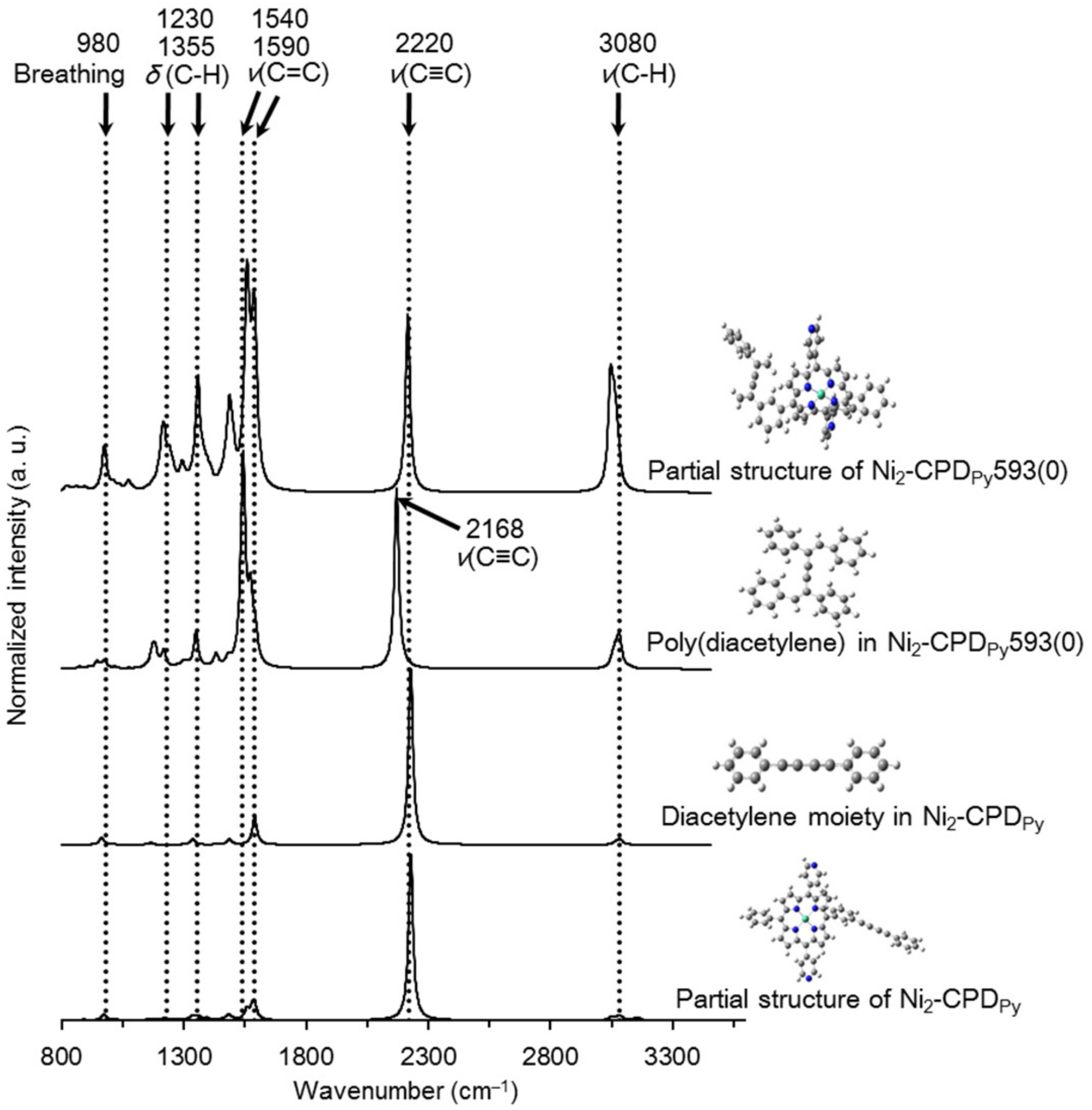

Supplementary Figure 12. Simulated Raman spectra. *ν*: stretching vibration. *δ*: out-of-plane vibration. Breathing: ring breathing. All the spectra were normalized by the peak intensity of *ν*(C≡C).

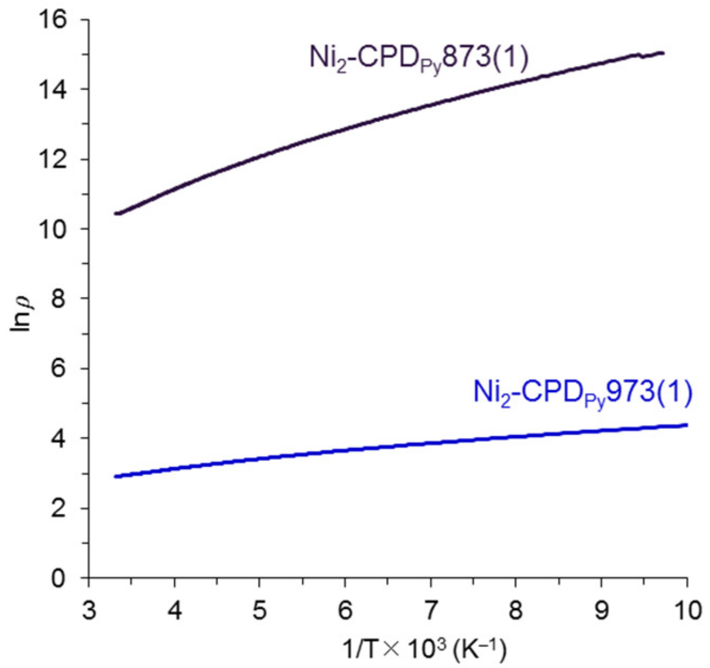

Supplementary Figure 13. Temperature dependence of resistivity (ρ [Ω cm]) for carbonized samples.

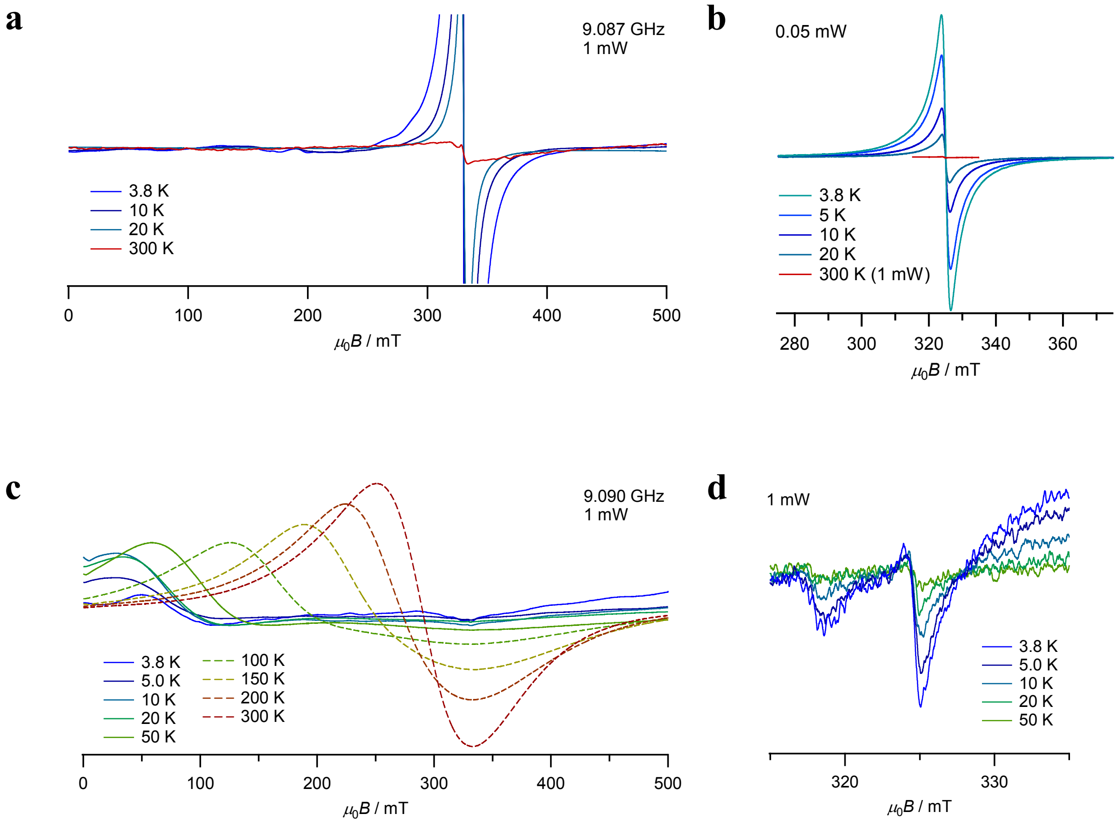

Supplementary Figure 14. X-band EPR spectra of carbonized samples. a, $Ni_2CPD_{Py}873(1)$ with the modulation field of 1.0 mT. Peaks at the temperatures of 3.8-20 K were saturated. At 300 K, the spectra is almost silent. **b**, $Ni_2CPD_{Py}873(1)$, 0.3 mT. The microwave power was decreased to avoid the saturation at 3.8-20 K. **c**, $Ni_2CPD_{Py}973(1)$, 1.0 mT. **d**, $Ni_2CPD_{Py}973(1)$, 0.3 mT. The amplifier gain was increased x10 times and the spectra was plotted in the x10 scale.

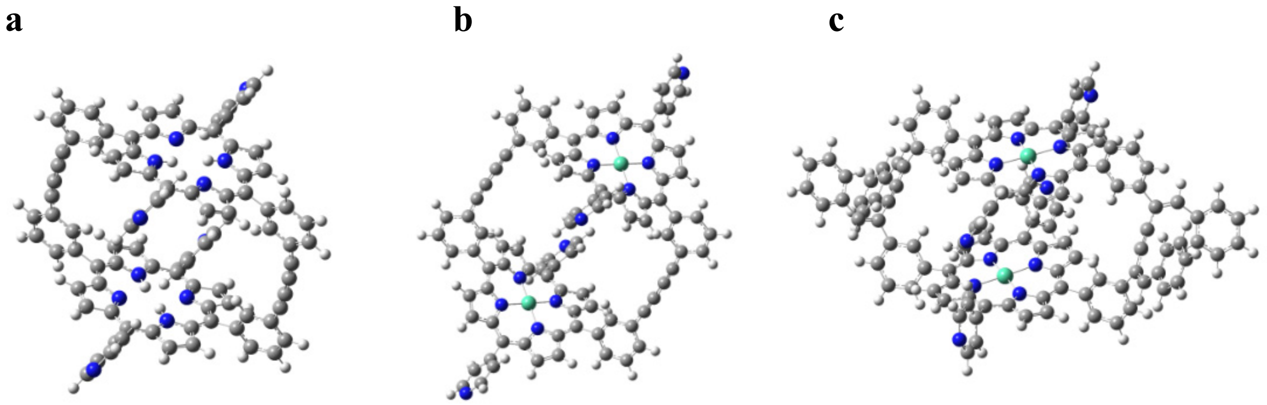

Supplementary Figure 15. The model structures used for the calculation of XPS spectra. a, H_4-CPD_{Py}. **b**, Ni_2-CPD_{Py}. **c**, Ni_2-CPD_{Py}593(0). These structures were subjected to structure optimization prior to the calculation.

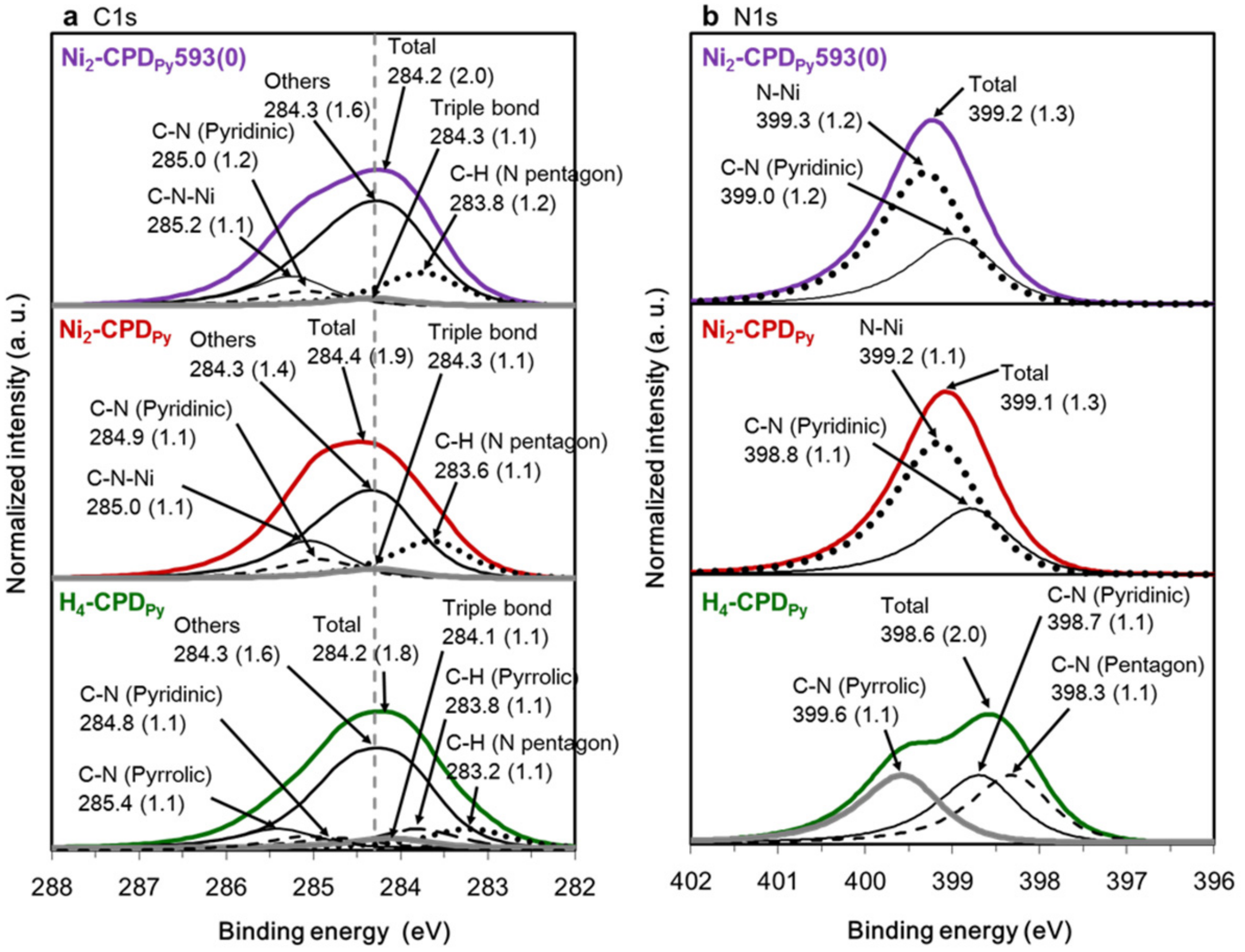

Supplementary Figure 16. Simulated C1s and N1s XPS spectra. a, C1s spectra. **b**, N1s spectra. Numbers in front of the parenthesis indicate binding energy in eV. Numbers inside the parentheses indicate FWHM of spectra. FWHM of 1.1 eV means the presence of one type of bonding state or similar bonding states.

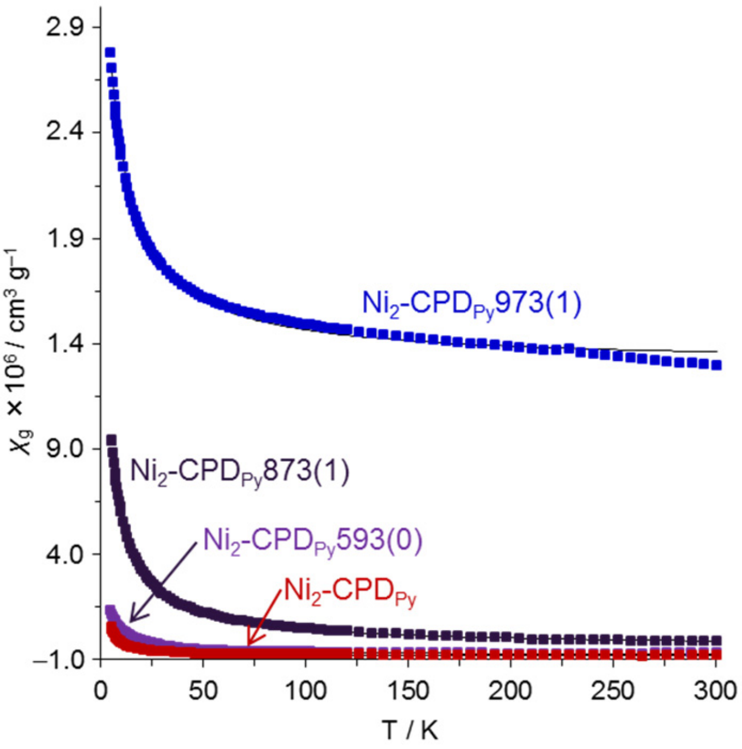

Supplementary Figure 17. Magnetic Susceptibilities. The data was fitted using the Curie-Weiss law $\chi_g = C / (T - \theta) + \chi_p$ where C is the Curie constant, θ is the Weiss temperature and χ_p is the temperature independent susceptibility. For Ni_2-CPD_{Py}, Ni_2-CPD_{Py}593(0) and Ni_2-CPD_{Py}873(1), χ_p was fixed to zero. See the text for the fitting parameters. The resulting fitting curves are shown by solid lines.

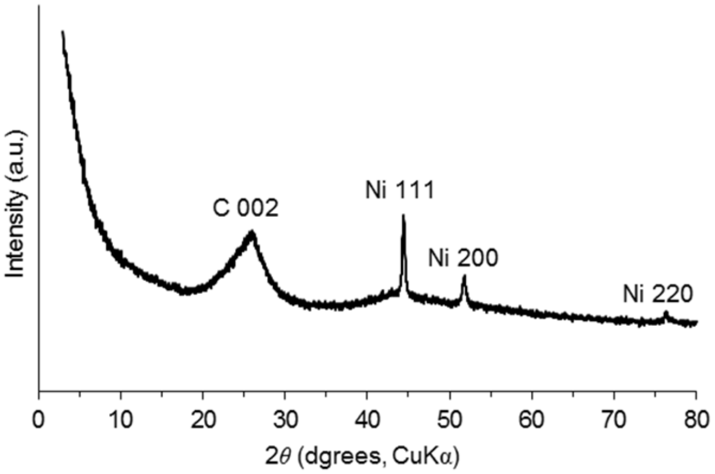

Supplementary Figure 18. PXRD pattern of Ni-TPP973(1). The measurement was carried out with an X-ray diffractometer (Rigaku, MiniFlex600) with Cu Kα radiation generated at 40 kV and 15 mA. There is a broad peak around 20-30°, corresponding to carbon (002) plane, and this indicates the development of graphene stacking structures in the carbonaceous matrix of this sample. Sharp peaks at 44.5°, 51.8°, and 76.4° are ascribed to Ni (111), (200), and (220) planes, respectively. Thus, it is found that some of Ni–N bonds are broken and Ni metal is formed. As is found in this case, it has been impossible to preseve the Ni–N_4 structure by simple carbonization of conventional Ni-based complexes. Ni-TPP973(1) does not show CO_2 reduction catalysis, and this clearly indicates that the presence of the Ni–N_4 unit in the carbonaceous framework is essential for this electrocatalysis.

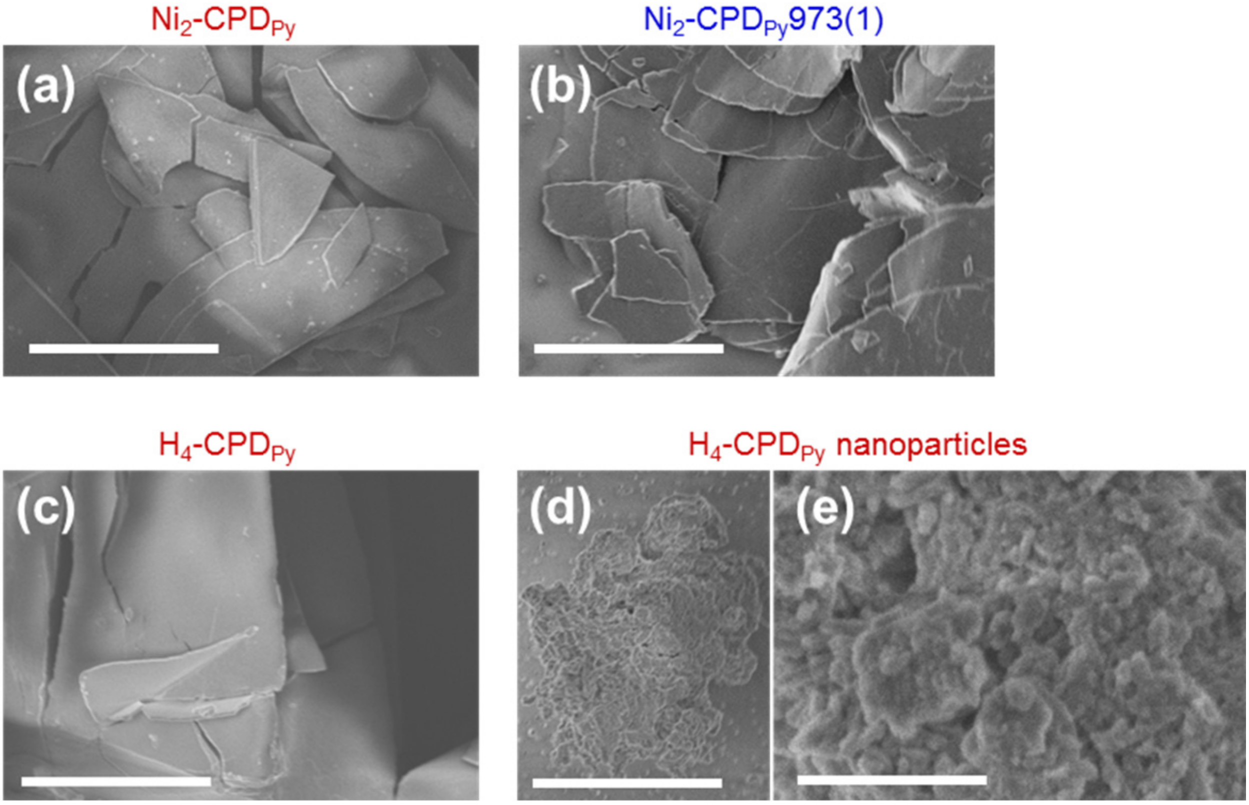

Supplementary Figure 19. SEM images of a, Ni$_2$-CPD$_{Py}$, b, Ni$_2$-CPD$_{Py}$973(1), c, H$_4$-CPD$_{Py}$, and d,e, H$_4$-CPD$_{Py}$ nanoparticles. Scale bars are 5 μm in (**a-d**), and 500 nm in (**e**). Ni$_2$-CPD$_{Py}$ is plate-like organic crystal (**a**), and the exterior shape is retained in OCF, Ni$_2$-CPD$_{Py}$973(1) (**b**), because the carbonization process proceeds in a solid phase. Hence, the exterior shape of OCFs can be controlled by the shape of their parent crystals. Here we show an example of the shape control in H$_4$-CPD$_{Py}$. While H$_4$-CPD$_{Py}$ is also a plate-like crystal (**c**), it is possible to form nanoparticles by the conventional reprecipitation method[57] (**d,e**). The H$_4$-CPD$_{Py}$ nanoparticles were prepared simply by injecting a H$_4$-CPD$_{Py}$/DMSO solution into a copious amount of water.

Supplementary Table 1. Parameters obtained by EXAFS mesurement.

Sample	N coordination number	*R* (Å)	*DW*[a] (Å)
Ni_2-CPD_{Py}	4.0	1.93	0.053
Ni_2-CPD_{Py}873(1)	3.8	1.86	0.060
Ni_2-CPD_{Py}973(1)	3.4	1.86	0.073

[a] Debye-Waller factor.

Supplementary Table 2. Porosity of Ni_2-CPD_{Py} and carbonaceous materials.

Sample	BET surface area ($m^2\ g^{-1}$)	V_{N2} ($cm^3\ g^{-1}$)	V_{CO2} ($cm^3\ g^{-1}$)
Ni_2-CPD_{Py}	n.m.[a]	n.m.	0.04
Ni_2-CPD_{Py}873(1)	48	0.08	0.11
Ni_2-CPD_{Py}973(1)	14	0.03	n.m.

[a] n.m.: Not measured.

As shown in Fig. 2g, OCF slightly shrinks from 873 to 973 K, and therefore, the porosity decreases at a higher temperature.

Supplementary Methods

PXRD analysis. The transmission method was applied to record the PXRD patterns (glass capillary diameter = 0.5 mm). Ni_2-CPD_{Py} and H_4-CPD_{Py} PXRD patterns were measured on a Rigaku SmartLab diffractometer (Cu $K\alpha_1$ radiation, 45 kV, 200 mA, 1.540593 Å) monochromatized by a Ge(111) Johansson-type crystal monochromator. Powder diffraction data were collected between 2–60° (2θ) with a 0.01° step for Ni_2-CPD_{Py} and 2–75° with a 0.007° step for H_4-CPD_{Py}. The PXRD patterns of the other samples were measured with a SPring-8 BL19B2 beamline (synchrotron X-ray beam, 0.99960 Å). The powder diffraction data were collected between 2–70° (2θ) with a 0.01° step on a diffractometer equipped with a blue imaging plate detector. The data collection time was 50 min for Ni_2-CPD_{Py}593(0) and 5 min for the other samples. The measured diffraction peaks were indexed by N-TREOR[28] and ITO13[30] for Ni_2-CPD_{Py} and Ni_2-CPD_{Py}593(0), respectively. The crystal structure was determined using the direct space method with the parallel tempering algorithm[29] followed by the Rietveld refinement[31]. All crystal structures were analysed using integrated X-ray powder diffraction software (PDXL Version 2.3.1.0, Rigaku). The structure refinement was carried out with a restraint function available in PDXL. The restraint parameters were taken from the Cambridge Crystallographic Data Centre (CCDC) by using the Mogul software. Since PDXL is not equipped with a structure refinement tool using a riding model, the positions of hydrogen atoms were determined by the restraint function in which the restraint parameters were optimized to achieve the similar result to that obtained by the riding model. Hydrogen atoms very little contribute to the PXRD results, and their positions can be refined almost automatically by the restraint function, after the determination of the positions of the non-hydrogen atoms. Hence, the result becomes almost the same as that by the riding model.

Construction of a possible structure model for Ni_2-CPD_{Py}873(1)

A possible structure model for Ni_2-CPD_{Py}873(1) was built by using the BIOVIA Materials Studio. From PXRD (Fig. 2g and Supplementary Fig. 8) and TEM/STEM (Fig. 2k, m and Supplementary Fig. 9) analyses, it can be concluded that the position of Ni is not very much changed during the carbonization from Ni_2-CPD_{Py}593(0) to Ni_2-CPD_{Py}873(1). Very high carbonization yield (Fig. 1b) together with a good retention of N and Ni species (Table 1 and Fig. 4) suggest that the structure change during the carbonization is not drastic. XAFS analysis (Fig. 5) indicates the preservation of Ni-N_4 unit in Ni_2-CPD_{Py}873(1). The decrease of H by the carbonization (Table 1), the black colour and the Raman spectrum of Ni_2-CPD_{Py}873(1) (Fig. 3a) revealed the formation of carbonaceous framework (polycyclic aromatic structure). TPD results (Supplementary Fig. 1b and c) suggest that

desorption occurs mainly from pyridyl and phenyl groups, and probably also from polydiacetylene backbones. Thus, it is assumed that porphyrin moieties of Ni_2-CPD_{Py}593(0) are unchanged and that other moieties turn into carbonaceous frameworks to crosslink the porphyrin moieties. Additionally, pyridinic and pyrrolic structures are introduced into the carbonaceous framework according to the XPS result (Fig. 4b). Probable minor structures are not taken into consideration: a small amount of oxygen probably introduced in defect sites, and dangling bonds suggested by EPR. The overall C/N/Ni/H ratio of the model is adjusted to almost equal to the experimental result (Table 1). Specifically, a supercell (2a×b×2c) based on the crystal structure of Ni_2-CPD_{Py}593(0) is first defined, and the structure included in the supercell is modified by hand to prepare an initial structure for the Ni_2-CPD_{Py}873(1) model, in accordance with the above assumption. Finally, the initial structure was optimized by the Forcite Plus (force field: Universal) under a periodic boundary conditions in the supercell. Upon the structure optimization, the positions of the porphyrin moieties are slightly moved, but the (020) plane is still well retained. Nevertheless, the positions of Ni atoms inside the (020) plane became random after the structure optimization, and this accords to the results shown in Supplementary Fig. 9d.

Simulation of Raman spectra

The calculations for simulating Raman spectra were conducted using B3LYP/6–31 g(d) integral = grid = ultrafine of the Gaussian 09 software[35]. The model structures used for the calculation are shown in Supplementary Fig. 11. After the structures were optimized, vibrational analyses for simulated Raman spectra using the keyword of freq = Raman were conducted. Charges and spin multiplicities were set as 0 and 1, respectively. The minimum full width at half maximum (FWHM) was set as 24 cm^{-1} and Lorentzian function was applied to calculate Raman spectra.

Simulation of XPS spectra

The calculations for simulating XPS spectra were conducted using B3LYP/6–31 g(d) integral = grid = ultrafine of the Gaussian 09 software[35]. The B3LYP/6–31g(d) level was selected on the basis of the prior works[40-44]. The structures of H_4-CPD_{Py}, Ni_2-CPD_{Py}, and Ni_2-CPD_{Py}593(0) (Supplementary Fig. 15) were subjected to structure optimization, and used for the calculation of XPS spectra. Population analyses for simulated XPS spectra using the keyword of pop = full gfprint were conducted using the optimized structures. Charges and spin multiplicities were set as 0 and 1, respectively. The orbital energies were converted into binding energies using the previously reported scaling factors[40-44]. The FWHM of spectra was set as 1.1 eV for one type of bonding state. Asymmetric Voigt function was applied to simulate XPS spectra.

Evaluation of selective CO_2 reduction into CO

The activities of Ni_2-CPD_{Py}973(1) and reference samples for CO_2 reduction reaction were evaluated based on the method reported elsewhere[55]. Briefly, the sample was mixed with Nafion solution (5%, Du Point, Corp.) dissolved with water, and the mixture was sonicated to form a catalyst ink. The ink was then dropped on a glassy carbon plate (2 cm^2) to fabricate a catalyst layer. The amount of loaded catalysts and Nafion was controlled to be 55 and 0.13 μg cm^{-2}, respectively. No conductive additive was used to evaluate the effect of sample conductivity. For reference, zeolite-templated carbon (ZTC), Ni_2-CPD_{Py}, and 5,10,15,20-tetraphenyl-21H,23H-porphine nickel(II) carbonized at 973 K for 1 h [Ni-TPP973(1)] were used.

An electrochemical measurement system (HZ-5000, Hokuto Denko) was used for the electrochemical measurements in a double-chamber electrochemical cell, in which the chambers were separated by a Nafion membrane, at room temperature. The counter and the reference electrodes were a platinum wire and an Ag/AgCl electrode, respectively. All potentials were referred to the RHE. Prior to the measurements, 0.1 M $KHCO_3$ electrolyte was saturated with CO_2, and the CO_2 reduction reaction was carried out. The generated gas products were quantitatively measured with a gas chromatography mass spectrometer (GCMS-QP 2010 Plus, Shimadzu) with micropacked ST MP-01 column (Shincarbon-ST 80/100 mesh)[55].
